# Catalytically inactive Cas9 attenuates DNA end resection: A potential application for region-restricted random mutagenesis

**DOI:** 10.1016/j.isci.2025.112702

**Published:** 2025-05-20

**Authors:** Suchin Towa, Satoshi Okada, Takashi Ito

**Affiliations:** 1 Department of Biochemistry, Kyushu University Graduate School of Medical Sciences, 3-1-1 Maidashi, Higashi-ku, Fukuoka 812-8582, Japan

**Keywords:** Techniques in genetics, Molecular genetics, Biological sciences research methodologies, Model organism

## Abstract

Gene duplication followed by sequence diversification is a key driver of innovation in genome evolution. To mimic this process in genome engineering, a method for region-restricted mutagenesis is needed to selectively mutate one copy of a duplicated gene. Notably, regions flanking a double-strand break (DSB) become hypersensitive to mutagens due to end resection, which converts them into single-stranded DNA (ssDNA). Blocking end resection could, therefore, confine hypermutation to a limited region. To achieve this, we investigated a catalytically inactive variant of *Streptococcus pyogenes* Cas9 (d*Sp*Cas9) and demonstrated its ability to attenuate end resection in the budding yeast *Saccharomyces cerevisiae* using ssDNA-specific quantitative PCR, live-cell imaging, and Southern blot analysis. By leveraging the bisulfite sensitivity of ssDNA, we further validated the concept of DSB-coupled, d*Sp*Cas9-mediated region-restricted mutagenesis. We anticipate that d*Sp*Cas9-mediated modulation of end resection at induced DSB sites will have valuable applications in both genome engineering and mechanistic studies.

## Highlights

• Positioning dCas9 near a double-strand break (DSB) inhibits end resection
• The end resection machinery is stalled at the dCas9-binding site
• dCas9 can limit the region susceptible to DSB-coupled bisulfite mutagenesis

## Graphical abstract

**Figure:**
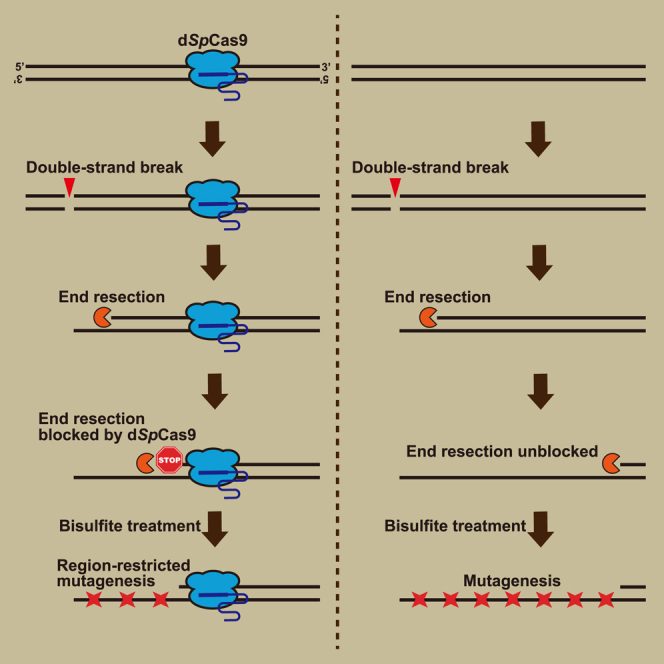

## Introduction

Gene duplication critically contributes to evolution and adaptation, not only by increasing gene dosage but by generating functionally differentiated paralogs through subsequent sequence diversification.^1^ Copy number variants are widespread in current human populations, with some contributing to disease pathogenesis. Accordingly, synthetic approaches that mimic evolution by gene duplication have gained increasing attention. For instance, we and others have developed methods to induce gene duplication/amplification using Cas9 mutants,^2^^,^^3^^,^^4^^,^^5^ which should enhance the dosage of genes within duplicated/amplified regions. To achieve sequence diversification, several methods for region-specific mutagenesis can be employed. These include cytoplasmic error-prone DNA polymerases that function orthogonally to host nuclear enzymes, Cas9 nickase-mediated recruitment of error-prone DNA polymerases to targeted regions, and T7 RNA polymerase-driven delivery of base editors to genes of interest, as recently reviewed.^6^ As a potential alternative to these methods, we became interested in double-strand break (DSB)-coupled regional mutagenesis, which recapitulates kataegis—a characteristic localized hypermutation observed in cancer genomes^7^—by inducing DSB in the presence of chemical mutagens or APOBEC cytidine deaminases.^8^^,^^9^

How can DSBs be coupled to mutagenesis? DSB repair mechanisms consist of two main categories: classical non-homologous end-joining (c-NHEJ) and homology-directed repair (HR). The latter includes diverse pathways, such as non-crossover generating synthesis-dependent strand annealing (SDSA), crossover/non-crossover generating pathway involving double Holliday junction (dHJ, originally referred to as DSB repair), break-induced replication (BIR), and single-strand annealing (SSA).^10^ In vertebrate cells, c-NHEJ remains active throughout the cell cycle and serves as the primary DSB repair pathway.^11^ Conversely, SDSA, dHJ, and BIR are primarily employed during the S phase of the cell cycle, using the unbroken sister chromatid as a template for precise DNA repair. While several studies have identified c-NHEJ as the dominant repair mode in mammalian cells in the G2 phase, HR is the preferred pathway for DSB repair in G2 cells of the budding yeast *Saccharomyces cerevisiae*.^12^^,^^13^^,^^14^ In contrast to c-NHEJ, HR relies on the generation of single-stranded DNA (ssDNA) through end resection. In *S. cerevisiae*, an endonuclease-induced DSB undergoes extensive resection, extending up to 50 kb from each end within 12 h when the DSB is unrepairable.^15^ In contrast, in repairable systems, the extent of resection is significantly shorter, typically ranging from less than 1–10 kb.^16^^,^^17^ Importantly, the phenomenon of kataegis is driven by the heightened susceptibility of ssDNA to mutagenic agents. Therefore, by blocking the progression of end resection at a specific site of interest, the spread of hypermutation could be regulated and we refer to this strategy as “controlled kataegis.”

How can end resection be blocked? It has been demonstrated that the progression of the end resection machinery is inhibited or even halted when it encounters challenging structures such as interstrand DNA crosslinks, bulky adducts, densely packed nucleosome arrays,^18^ and Ty transposons.^19^ As we cannot control the occurrence of these natural barriers, we sought to introduce artificial roadblocks to impede the resection process at specific genomic loci of interest, with the goal of achieving controlled kataegis. It should be also noted that such artificial attenuation of end resection can influence the repair outcome. For example, exposing repetitive elements on both sides of a DSB is expected to promote SSA at the expense of SDSA and dHJ. Conversely, blocking end resection before it reaches these repetitive elements would prevent SSA and redirect repair toward SDSA or dHJ.

With these considerations in our minds, we turned to the CRISPR-Cas (clustered regularly interspaced short palindromic repeats and CRISPR-associated proteins) systems as a promising candidate for the programmable, site-specific blocking agent of end resection. Among these enzymes, Cas9 from *Streptococcus pyogenes* (*Sp*Cas9), Cas9 from *Staphylococcus aureu*s (*Sa*Cas9), and Cas12a from *Acidaminococcus* sp. (*As*Cas12a), along with their respective derivatives, have been successfully used in a variety of applications.^20^^,^^21^^,^^22^^,^^23^^,^^24^ A specific derivative of *Sp*Cas9, known as catalytically inactive or dead *Sp*Cas9 (d*Sp*Cas9), has been engineered to eliminate its endonuclease catalytic function through two point mutations, while retaining its ability to bind to target DNA sequences through a protospacer adjacent motif (PAM) and a guide RNA sequence.^25^^,^^26^ The utility of d*Sp*Cas9 has been demonstrated in a variety of applications, including live-cell imaging of genomic loci of interest and activation/inactivation of target genes.^27^^,^^28^ Targeted gene inactivation by d*Sp*Cas9 involves two distinct, but not mutually exclusive, mechanisms: (1) inhibition of transcription initiation by steric hindrance and/or recruitment of epigenetic modulators and (2) inhibition of transcription elongation by blocking the progression of elongating RNA polymerase complex. The latter mechanism suggests that d*Sp*Cas9 can act as a programmable *in vivo* roadblock to impede the progression of machinery moving along the genomic DNA other than the transcriptional machinery. Indeed, we have shown that d*Sp*Cas9 inhibits the progression of DNA replication forks at a targeted site in *S. cerevisiae* to induce focal genomic instability.^29^

In this study, we first examined d*Sp*Cas9 using three distinct assays and demonstrated its ability to attenuate the progression of end resection machinery in *S. cerevisiae*. Based on this finding, we then validated the concept of region-restricted mutagenesis termed controlled kataegis in a yeast model system using bisulfite as the mutagen.^30^

## Results

### Design of ssDNA-specific qPCR assay

Since the primary product of end resection is ssDNA, its amount has to be accurately quantified to determine whether, and to what extent, d*Sp*Cas9 affects the progression of end resection. Therefore, we decided to use an ssDNA-specific qPCR protocol, which involves the digestion of genomic DNA with a restriction enzyme prior to the amplification of a DNA fragment containing the enzyme recognition site, referred to hereinafter as the amplicon.^31^ In this study, we used SphI because it cleaves its target sites in double-stranded DNA (dsDNA) but not in ssDNA. Accordingly, SphI digestion selectively eliminates SphI site-containing amplicons in dsDNA form (amplicons not reached by end resection) and leaves those in ssDNA form (those reached by end resection), allowing accurate quantification of the latter.

**Figure 1:**
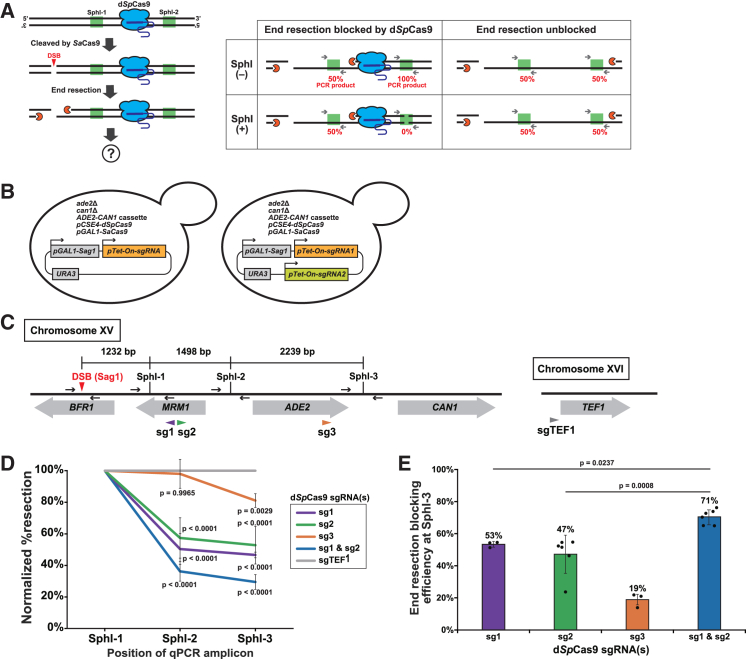
Single-stranded DNA-specific qPCR provides evidence for d*Sp*Cas9-mediated end resection blockage (A) Schematic representation of the ssDNA-specific qPCR assay used to quantify the progression of end resection from a DSB generated by *Sa*Cas9. Blue icons represent d*Sp*Cas9 molecules bound to the target genomic sequence. Green rectangles indicate qPCR amplicons containing a single SphI site, with gray arrows indicating forward and reverse primers. Orange icons indicate the end resection machinery. (B) Schematic representation of the strains used in this experiment. Sag1 is a sgRNA designed for *Sa*Cas9-mediated DSB generation, with its expression driven by the *GAL1* promoter (*pGAL1-Sag1*). In contrast, sgRNAs designed for targeting d*Sp*Cas9 are expressed under the control of the *Tet-On* promoter system (*pTet-On-sgRNA*). Each sgRNA is flanked by hammerhead and HDV ribozymes to enable autonomous excision from the primary transcript. The left panel shows a strain harboring a single d*Sp*Cas9 sgRNA, while the right panel shows a strain harboring a pair of d*Sp*Cas9 sgRNAs. (C) Schematic representation of the genomic locus used in this study. In the left panel, the DSB site generated by *Sa*Cas9(Sag1) is indicated by a red arrowhead. The target sites of three d*Sp*Cas9 sgRNAs (sg1, sg2, and sg3) are represented by purple, green, and orange arrowheads, respectively. The positions of three qPCR amplicons (SphI-1, SphI-2, and SphI-3), each containing a single SphI site, are shown as black vertical bars accompanied by pairs of horizontal arrows representing the qPCR primers. In the right panel, a gray arrowhead represents the target site of a d*Sp*Cas9 sgRNA (sgTEF1) on chromosome XVI, used in the DSB reference strain. (D) Normalized percentage of resection at three qPCR amplicon sites in five strains expressing distinct sgRNA(s), measured 4 h after DSB induction. Data are represented as mean ± standard deviation (SD) (*n* = 3 or 6 biological replicates). Statistical significance was assessed using Dunnett’s test for the SphI-2 and SphI-3 sites in strains harboring sgRNA(s) (sg1 & sg2, sg1, sg2, or sg3), compared to the DSB reference strain carrying a control sgRNA (sgTEF1). (E) End resection blocking efficiency of four strains at the SphI-3 site. Data are represented as mean ± SD (*n* = 3 or 6 biological replicates). Mean values are indicated above the bars, and individual dots represent biological replicates for each strain. Statistical significance was assessed using Dunnett’s test, comparing each strain harboring a single sgRNA (sg1 or sg2) to the strain harboring both sgRNAs (sg1 and sg2).

The rationale for our strategy to study the effects of d*Sp*Cas9 binding on end resection is illustrated in Figure 1A. We use an inducible DSB site and its flanking SphI sites. We refer to the SphI sites the most proximal and the second most proximal to the DSB site as SphI-1 and SphI-2, respectively. After targeting d*Sp*Cas9 to a site between SphI-1 and SphI-2, the DSB is induced so that the end resection machinery collides with the d*Sp*Cas9 placed on its track. If the d*Sp*Cas9 does not inhibit the progression of the machinery at all, both SphI-1 and SphI-2 amplicons should be converted to ssDNA, resulting in a qPCR yield of 50% compared to the amount obtained from the intact dsDNA, irrespective of SphI digestion. Conversely, if the d*Sp*Cas9 completely blocks progression, the SphI-1 amplicon should become single-stranded while the SphI-2 amplicon remains double-stranded. Accordingly, the yield of the SphI-1 amplicon will be 50% irrespective of SphI digestion, while that of the SphI-2 amplicon will be 0% and 100% with SphI and mock digestion, respectively. We can calculate the blocking efficiency by comparing the yields of the SphI-2 amplicon in the presence and absence of d*Sp*Cas9 bound to its upstream site.

To implement this rationale, we constructed a reporter strain by inserting an *ADE2-CAN1* cassette into the *his3* locus on chromosome XV in an *ade2*Δ *can1*Δ strain (Figure 1B). We selected a *Sa*Cas9-inducible DSB site in *BFR1* and used three SphI sites to design qPCR amplicons (Figure 1C). In this strain, the expression of the cutter (*Sa*Cas9) is driven by the *GAL1* promoter, while the potential blocker (d*Sp*Cas9) is constitutively expressed from the *CSE4* promoter. The strain also contains a centromeric plasmid that expresses a single guide RNA (sgRNA) for *Sa*Cas9 by the *GAL1* promoter and one or two sgRNAs for d*Sp*Cas9 by the *Tet-On* promoter (Figure 1B).

In practice, we first use doxycycline (Dox) to induce sgRNAs that recruit d*Sp*Cas9 to the target sites: sg1 and sg2 to sites between SphI-1 and SphI-2; sg3 to a site between SphI-2 and SphI-3; and sgTEF1 to an upstream site proximal to *TEF1* on chromosome XVI (Figure 1C). Next, we use galactose (Gal) to co-induce the *Sa*Cas9 protein and its sgRNA (Sag1) to introduce a DSB in *BFR1* (Figure 1C). We also constructed a similar set of strains using en*As*Cas12a and its CRISPR RNA (crRNA) termed cr1 to induce another DSB in *BFR1* (see below). The strains used in this study follow the nomenclature format “SagX_sgX” or “crX_sgX”, where the first segment indicates the sgRNA for *Sa*Cas9 or crRNA for en*As*Cas12a to induce DSB, while the second segment indicates the sgRNA for d*Sp*Cas9 to block end resection (see Table S1 for details).

### ssDNA-specific qPCR evidence for d*Sp*Cas9-mediated end resection blockage

To determine the optimal time-point for evaluation, we first assessed the time course of DSB formation in the reporter strains following Gal induction of *Sa*Cas9. Using a qPCR primer pair sandwiching the DSB site (Figure 1C), we quantified the frequency of DSB formation in the Sag1_sgTEF1 strain, which served as a DSB reference strain with no d*Sp*Cas9 bound to the reporter locus, at 0, 2, 4, 6, and 8 h after Gal addition. Although DSB induction remained modest at 2 h (17%), it plateaued at 4, 6, and 8 h (48%, 55%, and 58%, respectively) (Figure S1A). Three other strains with d*Sp*Cas9 targeting the reporter locus (Sag1_sg1&sg2, Sag1_sg2, and Sag1_sg3) showed similar patterns (Figure S1A). Therefore, we chose the 4-h induction period for ssDNA-specific qPCR analysis.

We evaluated the resection efficiencies at SphI-1, SphI-2, and SphI-3 after 4 h of DSB induction in four test strains (Sag1_sg1, Sag1_sg2, Sag1_sg3, and Sag1_sg1&sg2) and the DSB reference strain (Sag1_sgTEF1) (Figures 1B and 1C; Table S1). After calculating the resection efficiencies, we performed two normalizations. First, we normalized the resection efficiencies at SphI-2 and SphI-3 to that at SphI-1, which should be unaffected by d*Sp*Cas9 because there was no d*Sp*Cas9 bound to the region between the DSB and SphI-1 (Figure 1C). Second, we normalized the resection efficiency at each SphI site in the test strains to that in the reference strain, which should have the natural end resection pattern because there was no d*Sp*Cas9 bound to the reporter locus. This normalization should eliminate the variance introduced by the different distances between the DSB and SphI sites.

With these normalization steps, we observed a reduction in the resection efficiency at SphI-2 compared to SphI-1 in the Sag1_sg1, Sag1_sg2, and Sag1_sg1&sg2 strains (Figure 1D). These results indicated that d*Sp*Cas9 bound between SphI-1 and SphI-2 inhibited the progression of the end resection machinery. Consistently, SphI-3 exhibited a resection efficiency comparable to that of SphI-2, reflecting the absence of roadblocks between these two sites (Figure 1D). In contrast, in the Sag1_sg3 strain, SphI-1 and SphI-2 showed a comparable resection efficiency, while SphI-3 showed a reduced efficiency compared to SphI-1 and SphI-2 (Figure 1D). This pattern indicated that d*Sp*Cas9 recruited by sg3 to the site between SphI-2 and SphI-3 inhibited the resection. These results are consistent with the patterns predicted from the scenario in which d*Sp*Cas9 inhibits the progression of the end resection machinery.

We can determine the blocking efficiency of end resection from the decrease in the normalized resection efficiency. The blocking efficiency obtained by targeting d*Sp*Cas9 to two sites simultaneously (double targeting) is expected to be higher than that obtained by targeting d*Sp*Cas9 to a single site individually (single targeting). Indeed, the blocking efficiencies at SphI-3 in the Sag1_sg1, Sag1_sg2, and Sag1_sg1&sg2 strains were 53%, 47%, and 71%, respectively (Figure 1E). If sg1 and sg2 independently allow d*Sp*Cas9 to attenuate end resection, the blocking efficiency with double targeting should be 75%, which is close to the observed value of 71%.

We conducted a similar experiment with strains using en*As*Cas12a for DSB induction (Figures S1B and S1C). During the 4-h induction, these strains exhibited a lower but stable frequency of DSB formation (12–15%) (Figure S1D). Analysis of normalized resection efficiencies at the three SphI sites revealed the expected pattern (Figure S1E). In both cr1_sg2 and cr1_sg1&sg2 strains, SphI-2 showed reduced and comparable levels of resection compared to SphI-1 and SphI-3, respectively (Figure S1E). As anticipated, the reduction was more pronounced with double targeting (cr1_sg1&sg2) than with single targeting (cr1_sg2) (Figure S1E). Conversely, the cr1_sg3 strain exhibited almost no and a significant reduction in end resection at SphI-2 and SphI-3, respectively, compared to SphI-1 (Figure S1E). The blocking efficiencies at SphI-3 in the cr1_sg2, cr1_sg3, and cr1_sg1&sg2 strains were 41%, 37%, and 60%, respectively (Figure S1F). For unknown reasons, the d*Sp*Cas9 recruited by sg3 showed a much higher blocking efficiency in the cr1_sg3 strain compared to the Sag1_sg3 strain (Figures 1E and S1F).

We should note that the progression of DSB repair events may have influenced the results of the ssDNA analysis described above. To eliminate such effects and focus specifically on end resection, we conducted a similar analysis in a *rad51*Δ strain lacking HR repair. This *rad51*Δ strain was generated by replacing the endogenous *RAD51* gene with *KanMX* (Figure S2A). After 4 h of DSB induction, the survival rate of *rad51*Δ cells dropped to less than half that of the wild-type *RAD51* strain (Figure S2B). Resection efficiency was analyzed in four derivatives of the *rad51*Δ strain: *rad51*Δ_Sag1_sg1&sg2, *rad51*Δ_Sag1_sg2, *rad51*Δ_Sag1_sg3, and *rad51*Δ_Sag1_sgTEF1. The results closely mirrored those observed in the wild-type strain (Figure S2C). At the SphI-3 site, end resection blocking efficiencies were 49% in the strain with d*Sp*Cas9 bound to sg2, 39% in the strain with d*Sp*Cas9 bound to sg3, and 62% in the strain with d*Sp*Cas9 bound to both sg1 and sg2 (Figure S2D). These findings indicate that d*Sp*Cas9 effectively blocks end resection in the absence of HR repair: the effects of on-going repair in the wild-type strain were minimal in the ssDNA analysis under our conditions.

Taken together, these qPCR results provide the first evidence that d*Sp*Cas9 attenuates the progression of end resection *in vivo*.

### Southern blot hybridization evidence for d*Sp*Cas9-mediated stalling of end resection

Note that the ssDNA-specific qPCR results do not definitively indicate that d*Sp*Cas9 directly stalls the progression of end resection at its binding site; it remains formally possible that d*Sp*Cas9 may indirectly affect the progression of the end resection machinery at a distance from its binding site. To address this, we designed a Southern blot hybridization experiment to assess the impact of d*Sp*Cas9 on DNA end resection progression (Figure S3A). For this purpose, we tracked the degradation of the 4.1-kb MluI-HindIII fragment, which spans the target site of *Sa*Cas9(Sag1), using a 180-mer strand-specific probe located near the HindIII site and complementary to the DNA strand that is degraded during end resection (Figure S3A). The *Sa*Cas9(Sag1)-induced DSB is expected to generate a 2.7-kb band, which should progressively degrade into a smear as end resection advances. Most importantly, if end resection stalls at the d*Sp*Cas9-binding site, a 1.3-kb band is anticipated to appear within the smear.

We extracted genomic DNA from the Sag1_sg1&sg2 and Sag1_sgTEF1 strains at 0, 2, 4, 6, and 8 h after DSB induction, digested the DNA with MluI and HindIII, and performed Southern blot hybridization with the strand-specific probe. The 4.1-kb band, representing the intact MluI-HindIII fragment, was present in all samples (Figure S3B), consistent with the observation that DSBs occur in ∼50% of cells (Figure S1A). Two hours after DSB induction, the 2.7-kb band appeared in both strains, indicating DSB formation without significant resection. This band gradually diminished over time, reflecting the progression of resection. Notably, the ∼1.3-kb band appeared in the Sag1_sg1&sg2 strain, but not in the Sag1_sgTEF1 strain, particularly at 4 h post-DSB induction (Figure S3B). Intriguingly, it gradually decreased after 4 h in a reproducible manner (Figure S3B). We presume this is due to d*Sp*Cas9 dissociating from its binding site, allowing end resection to resume. Indeed, in human cells, d*Sp*Cas9 has been shown to have a residence time of ∼206 min on perfectly matched sequences, indicating eventual dissociation.^32^^,^^33^ While specific studies on the transient nature of d*Sp*Cas9 binding to its target site in *S. cerevisiae* are, to our knowledge, unavailable, such dissociation is likely to occur in yeast as well.

Taken together, these results indicate that d*Sp*Cas9 directly stalls the progression of DNA end resection at its binding site, as anticipated.

### Design of ssDNA-specific live-cell imaging assay

**Figure 2:**
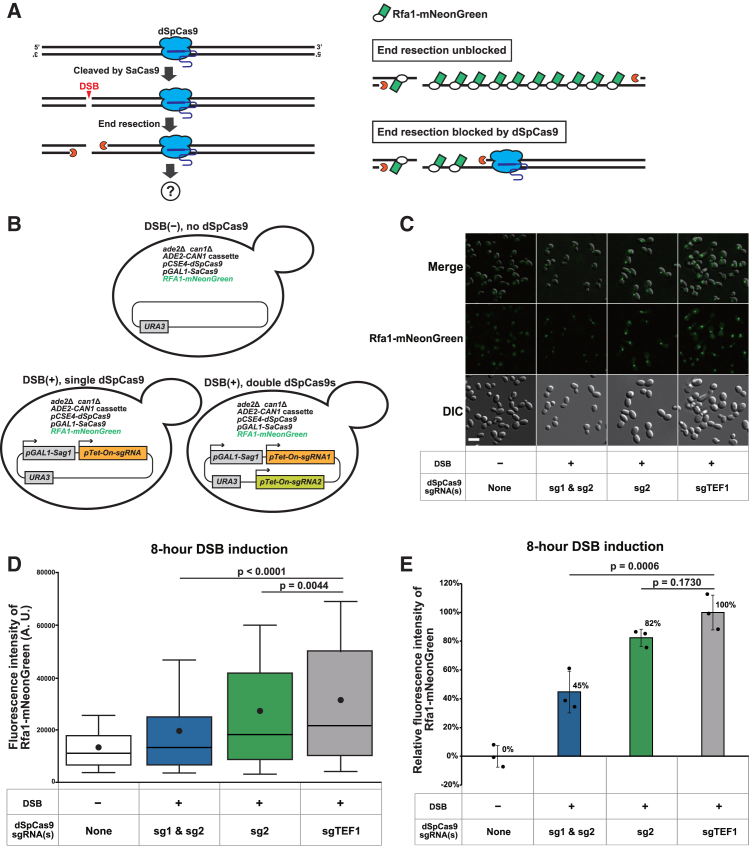
Live-cell imaging provides evidence for d*Sp*Cas9-mediated end resection blockage (A) Schematic representation of the ssDNA-specific live-cell imaging assay used to assess the effect of d*Sp*Cas9 on end resection. Blue icons represent d*Sp*Cas9 molecules bound to the target genomic sequence. Orange icons indicate the end resection machinery. White ovals indicate the ssDNA-binding protein Rfa1, while green rectangles represent the yellow-green fluorescent protein mNeonGreen fused to Rfa1. (B) Schematic representation of the strains used for live-cell imaging. The top panel shows the negative control strain without DSB generation by *Sa*Cas9 and without d*Sp*Cas9 binding (mNG_Sa_NC). The bottom left panel shows the strain harboring a single d*Sp*Cas9 sgRNA, while the bottom right panel shows the strain harboring a pair of d*Sp*Cas9 sgRNAs. In both strains shown in the bottom panels, DSBs are generated by *Sa*Cas9(Sag1). (C) Representative images of yeast cells under the indicated conditions, acquired 8 h after DSB induction. The images include Rfa1-mNeonGreen fluorescence (green), DIC images (grayscale), and merged overlays. Scale bar, 10 μm. (D) Boxplots showing the distribution of Rfa1-mNeonGreen fluorescence intensity in cells under the indicated conditions. Images were acquired 8 h after DSB induction. The DSB was generated by *Sa*Cas9(Sag1). Boxes represent the 25th and 75th percentiles, whiskers represent the 10th and 90th percentiles, the horizontal line inside the box indicates the median, and the black dot represents the mean. Statistical significance was evaluated using Steel-Dwass’s test, comparing strains carrying either a pair of sgRNAs (sg1 and sg2) or a single sgRNA (sg2) with the DSB reference strain harboring sgTEF1. (E) Relative fluorescence intensity of Rfa1-mNeonGreen in cells under the indicated conditions, measured 8 h after DSB induction. The DSB was generated by *Sa*Cas9(Sag1). Data are represented as mean ± SD (*n* = 3 biological replicates). Mean values are indicated at the upper right of the bars, and individual dots represent biological replicates for each strain. Statistical significance was assessed using Dunnett’s test, comparing strains harboring either a pair of sgRNAs (sg1 and sg2) or a single sgRNA (sg2) with the DSB reference strain harboring sgTEF1.

We next sought to confirm d*Sp*Cas9-mediated attenuation of end resection using an alternative assay to qPCR and Southern blot hybridization. To this end, we used our own microscopic method to quantify the amount of ssDNA using Rfa1, a subunit of the heterotrimeric ssDNA-binding protein complex replication protein A (RPA),^34^^,^^35^ fused to a bright yellow-green fluorescent protein, mNeonGreen.^36^ After DSB formation and subsequent end resection, Rfa1-mNeonGreen proteins accumulate on the ssDNA, allowing their detection by fluorescence microscopy to serve as an indicator of ssDNA levels in living cells. If DNA-bound d*Sp*Cas9 inhibits end resection from a DSB site, the amount of ssDNA should decrease, resulting in a concomitant reduction in the number of ssDNA-bound Rfa1-mNeonGreen proteins. As a result, the intensity of mNeonGreen fluorescence observed by fluorescence microscopy is expected to decrease (Figure 2A).

To implement this simple logic, we genetically tagged the endogenous *RFA1* gene with the *mNeonGreen* gene in the strains used for ssDNA-specific qPCR and designated the resulting strains mNG_Sag1/cr1_sgX (Figures 2B; Table S1). In addition, we constructed negative control strains lacking both Sag1/cr1 and sgX and designated them mNG_Sa/enAs_NC (Figures 2B; Table S1). It is important to evaluate the background fluorescence induced by physiological levels of DSBs using these control strains because the Rfa1-mNeonGreen fluorescence is not specific to the DSB at the site of interest.

### Live-cell imaging evidence for d*Sp*Cas9-mediated end resection blockage

To determine the optimal time-point for evaluation, we first examined the fluorescence intensity in the mNG_Sag1_sgTEF1 strain and a negative control strain of mNG_Sa_NC at 0, 2, 4, 6, 8, and 10 h after DSB induction. To eliminate the influence of background fluorescence, the fluorescence intensity of negative control (mNG_Sa_NC) was subtracted from that of the mNG_Sag1_sgTEF1 strain at each time point to obtain the background-subtracted fluorescence intensity of the mNG_Sag1_sgTEF1 strain. The intensity gradually increased from 4 h, reached its maximum at 8 h, and showed signs of decline at 10 h (Figure S4A), which might reflect the Rad52-mediated replacement of RPA with Rad51. Therefore, we induced DSBs for 8 h in the following experiments.

We assessed fluorescence intensity in four strains (mNG_Sa_NC, mNG_Sag1_sg1&sg2, mNG_Sag1_sg2, and mNG_Sag1_sgTEF1) before (0 h) and 8 h after DSB induction. All four strains exhibited comparable levels of weak fluorescence at 0 h, reflecting the background level of DSB formation independent of the induced cleavage by *Sa*Cas9 (Figure S4B). In contrast, these strains showed remarkable differences in the distribution of fluorescence intensities at 8 h (Figures 2C and 2D). As expected, the distribution showed the most pronounced and marginal upward shift in the mNG_Sag1_sgTEF1 and mNG_Sa_NC strains, respectively (Figures 2D and S4B). The upward shift was reduced in the order of mNG_Sag1_sg2, followed by mNG_Sag1_sg1&sg2: double targeting of d*Sp*Cas9 inhibited the increment of fluorescence more efficiently than single targeting. The cells with DSB induction exhibit a larger size compared to the negative control cells (no DSB induction) (Figure 2C, bottom panel). This increase in cell size should be attributed to the robust cell-cycle arrest triggered by DSBs, which allows the cell sufficient time for DNA repair and ensures the maintenance of genomic integrity.^37^^,^^38^

We also calculated the relative fluorescence intensity of the cells after the 8-h DSB induction. The strain with double targeting (mNG_Sag1_sg1&sg2) showed the lowest relative Rfa1-mNeonGreen fluorescence intensity among the three strains with DSB induction, followed by the strain with single targeting (mNG_Sag1_sg2). The strain with no d*Sp*Cas9 binding in the DSB-flanking regions (mNG_Sag1_sgTEF1) showed the highest relative intensity (Figure 2E).

We performed a similar analysis on strains using en*As*Cas12a with cr1 for DSB induction (Figure S4C). Unlike in mNG_Sag1_sgTEF1 (Figure S4A), the fluorescence intensity in the strain mNG_cr1_sgTEF1 continued to increase until 10 h after DSB induction (Figure S4D). Because the increase rate in fluorescence intensity from 8 h to 10 h decreased to almost one-third of that from 4 h to 8 h, we selected 8 h as the DSB induction time for the assay and observed a qualitatively similar pattern to that described above (Figures S4E and S4F).

Taken together, the results of the ssDNA-specific live-cell imaging assay consistently support the notion that d*Sp*Cas9 attenuates DNA end resection.

### Correlation between the ssDNA-specific qPCR and live-cell imaging results

**Figure 3:**
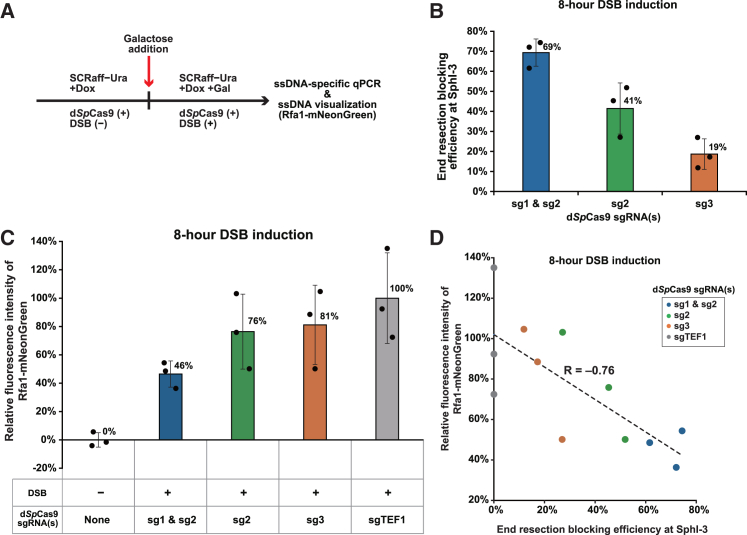
End resection blocking efficiency inversely correlates with Rfa1-mNeonGreen fluorescence intensity (A) Schematic representation of the experimental workflow to evaluate end resection blocking efficiency and Rfa1-mNeonGreen fluorescence intensity. SCRaff−Ura refers to synthetic complete medium containing 3% raffinose and lacking uracil. Expression of d*Sp*Cas9 sgRNA(s) is induced by the addition of Dox, while expression of *Sa*Cas9 and its sgRNA for DSB generation is induced by the addition of Gal. (B) End resection blocking efficiency at the SphI-3 site in cells with the DSB generated by *Sa*Cas9(Sag1) and d*Sp*Cas9 guided by the indicated sgRNAs. ssDNA-specific qPCR was performed 8 h after the DSB induction. Data are represented as mean ± SD (*n* = 3 biological replicates). Mean values are indicated at the upper right of the bars, and individual dots represent biological replicates for each strain. (C) Relative Rfa1-mNeonGreen fluorescence intensity in cells under the indicated conditions, measured 8 h after DSB induction. The DSB was generated by *Sa*Cas9(Sag1). Data are represented as mean ± SD (*n* = 3 biological replicates). Mean values are indicated at the upper right of the bars, and individual dots represent biological replicates for each strain. (D) Correlation between end resection blocking efficiency at the SphI-3 site and relative Rfa1-mNeonGreen fluorescence intensity in cells under different d*Sp*Cas9-bound conditions, measured 8 h after DSB induction. The DSB was generated by *Sa*Cas9(Sag1). Measurements were performed in triplicate for each strain.

We next investigated the correlation between the results of the ssDNA-specific qPCR and the live-cell imaging obtained from the same cell population. For this purpose, we performed a time-course experiment (Figure 3A) using the strains employed in the live-cell imaging assay and the mNG_Sag1_sg3 strain (Figures 1C and 2B).

The optimal duration for DSB induction was determined to be a period of 4–8 h and 8 h for the qPCR and live-cell imaging assays, respectively, as described above. We measured the resection efficiency at each SphI site 4, 6, and 8 h after DSB induction using the ssDNA-specific qPCR assay and calculated the blocking efficiencies at SphI-3 (Figures 3B, S5A, and S5B). At 8 h after induction, the blocking efficiencies at SphI-3 were 69% in the mNG_Sag1_sg1&sg2 strain, 41% in the mNG_Sag1_sg2 strain, and 19% in the mNG_Sag1_sg3 strain (Figure 3B). These results were consistent with those obtained in their parental strains (Figure 1E), confirming that mNeonGreen-tagging of Rfa1 had little to no effect on the end resection. The patterns of end resection blocking efficiencies at 4 and 6 h after DSB induction were qualitatively similar to those at 8 h (Figures 3B, S5A, and S5B), further confirming that DSB induction from 4 to 8 h is appropriate for the qPCR assay.

We also evaluated the relative fluorescence intensity of Rfa1-mNeonGreen at 4, 6, and 8 h after DSB induction. Since the optimal DSB induction time for the ssDNA-specific live-cell imaging assay was determined to be 8 h, it is not surprising that samples induced for 8 h showed the expected pattern, whereas those induced for 4 h or 6 h did not (Figures 3C and S5C).

We then performed Pearson’s correlation analysis between the blocking efficiencies obtained from the ssDNA-specific qPCR assay and the relative fluorescence intensity of Rfa1-mNeonGreen in the samples subjected to DSB induction for 8 h. The resulting correlation coefficient was −0.76 (Figure 3D).

These results collectively suggest that the ssDNA-specific live-cell imaging assay can serve as a versatile alternative or proxy for the ssDNA-specific qPCR assay.

### A potential application of d*Sp*Cas9 to region-restricted random mutagenesis

It is well known that ssDNA is more susceptible to mutagenesis than dsDNA due to the exposure of nucleobases.^39^^,^^40^^,^^41^^,^^42^ Previous studies have shown that DSBs induced in the presence of UV, alkylating agents, bisulfite, or APOBEC can lead to mutagenesis in their flanking regions, resembling kataegis or clustered mutations observed in cancer genomes.^8^^,^^9^^,^^30^^,^^43^^,^^44^^,^^45^ These results reflect that Rad51 mediates ssDNA invasion to initiate DSB repair, even in the presence of a certain level of mismatches between the ssDNA and its donor sequence, and that mismatches escaping mismatch repair can lead to mutations.^46^ Therefore, we hypothesized that d*Sp*Cas9 could be repurposed to control or restrict the region susceptible to experimentally induced kataegis: the DSB-distal side of the d*Sp*Cas9-binding site exhibits a lower mutation rate than the DSB-proximal side and is thus protected from kataegis. We termed this approach of region-restricted random mutagenesis controlled kataegis.

**Figure 4:**
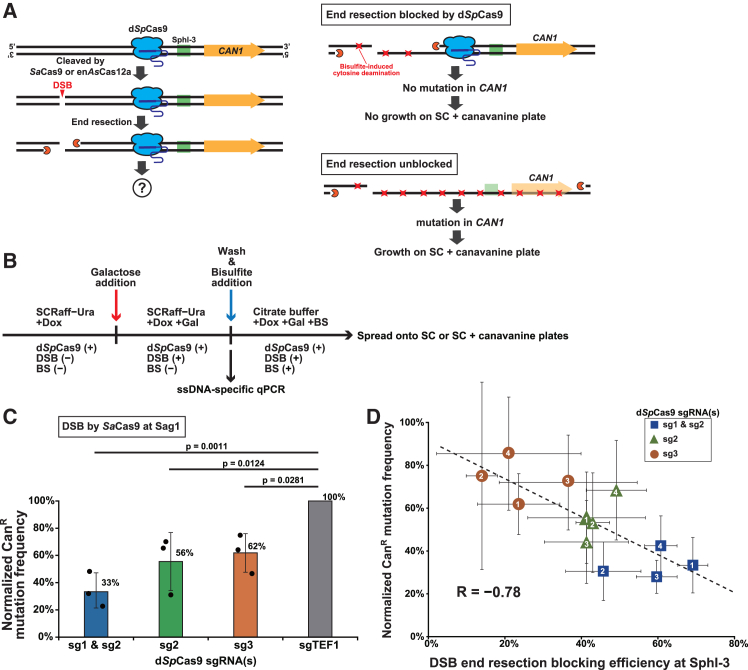
d*Sp*Cas9-mediated end resection blockage enables region-restricted random mutagenesis (A) Schematic representation of the genetic assay used to evaluate region-restricted random mutagenesis. Blue icons represent d*Sp*Cas9 molecules bound to the target genomic sequence. Green rectangles indicate the qPCR amplicon containing a single SphI site. Orange icons depict the end resection machinery. Orange arrows denote the *CAN1* gene. Red cross marks indicate the locations of bisulfite-induced base substitutions. (B) Schematic representation of the experimental workflow for the genetic assay of bisulfite-induced mutagenesis and the ssDNA-specific qPCR assay. Expression of d*Sp*Cas9 and its sgRNA(s) is induced by the addition of Dox, while expression of *Sa*Cas9/en*As*Cas12a and their corresponding sgRNA/crRNA for DSB generation is induced by the addition of Gal. “BS” stands for bisulfite. (C) Normalized Can^R^ mutation frequencies following bisulfite treatment in the four strains, each with the DSB generated by *Sa*Cas9(Sag1) and the indicated d*Sp*Cas9-bound condition. Data are represented as mean ± SD (*n* = 3 biological replicates). Mean values are indicated at the upper right of the bars, and individual dots represent biological replicates for each strain. Statistical significance was assessed using Dunnett’s test for each of the three strains compared to the DSB reference strain harboring sgTEF1. (D) Correlation between end resection blocking efficiency at the SphI-3 site and normalized Can^R^ mutation frequency in cells subjected to different DSB induction and d*Sp*Cas9-bound conditions. End resection blocking efficiencies at the SphI-3 site were measured by ssDNA-specific qPCR using samples collected 4 h after DSB induction. DSBs were generated by four distinct systems—*Sa*Cas9(Sag1), *Sa*Cas9(Sag2), en*As*Cas12a(cr1), and en*As*Cas12a(cr2)—indicated as 1, 2, 3, and 4 in the icons, respectively. Data are represented as mean ± SD (*n* = 3 biological replicates).

We performed a proof-of-concept experiment for controlled kataegis using the *CAN1* gene in the reporter strain. The rationale for this experiment is shown in Figure 4A. In the absence of d*Sp*Cas9-mediated end resection blockage, DSB induction results in the conversion of *CAN1* to ssDNA, rendering it highly susceptible to bisulfite-induced mutagenesis.^30^ Conversely, d*Sp*Cas9 bound to the region between the DSB and *CAN1* should protect the latter from end resection and mutagenesis. Since a fraction of *CAN1* mutations confers canavanine resistance (Can^R^), we can estimate the mutation frequency from the number of colonies appearing on canavanine-containing agar plates. Ideally, d*Sp*Cas9 binding would eliminate the appearance of Can^R^ colonies (Figure 4A).

We repurposed the same strains used in the ssDNA-specific qPCR assay for this experiment (Figure S6A). First, we optimized the bisulfite treatment conditions. After Dox-induced expression of sgRNAs for d*Sp*Cas9, we induced the DSB with Gal for 4 h and then treated the cells with or without 1% bisulfite in the presence of Dox and Gal for 2.5 h (Figure 4B). Under these conditions, survival rates were comparable between the presence and absence of 1% bisulfite and between the presence and absence of the targeted d*Sp*Cas9, whereas DSB induction significantly decreased the viability (Figure S6B).

We included two normalization steps: the mutation frequency of each strain was normalized to its DSB formation frequency as assessed by qPCR, and the mutation frequencies of the test strains were normalized to the DSB reference strain to facilitate comparison between different experiments.

Consequently, we determined the normalized Can^R^ frequencies in the bisulfite-treated Sag1_sg1&sg2, Sag1_sg2, and Sag1_sg3 strains to be 33%, 56%, and 62%, respectively, compared to the DSB reference strain (Sag1_sgTEF1) (Figure 4C). Thus, d*Sp*Cas9 resulted in an up to 3-fold reduction in the mutation rate on the DSB-distal side of its binding site. The double-targeting strain with the highest block efficiency exhibited the lowest mutation frequency on the DSB-distal side of the d*Sp*Cas9 binding site(s), consistent with the scenario that blocking end resection leads to protection from mutagenesis. This notion was further reinforced by a negative correlation of −0.78 between blocking efficiency at SphI-3 and normalized Can^R^ mutation rates observed in 12 strains with different combinations of four DSB sites (Sag1, Sag2, cr1, and cr2) and three d*Sp*Cas9-binding patterns (sg1&sg2, sg2, and sg3) (Figures 4D and S6A).

Since Cas9 cleavage occasionally induces large on-target deletions, some Can^R^ clones may arise not from bisulfite-induced point mutations, but from such deletions. We thus took advantage of the *ADE2* gene embedded between the DSB and *CAN1* reporter gene. Since *ADE2* is more proximal to the DSB site than *CAN1*, on-target deletions reaching *CAN1* inevitably include *ADE2*. The resultant *ade2*Δ Can^R^ colonies should turn red on canavanine-containing agar plates supplemented with a low concentration of adenine. In our test locus, only a minority of Can^R^ colonies exhibited the red color (Figures S6C and S6D), indicating that the effect of on-target deletion was negligible.

Note that the *CAN1* reporter can become single-stranded not only through end resection but also through transient R-loop formation associated with its transcription. End resection following DSB formation by *Sa*Cas9(Sag1) exposes the non-sense strand of the *CAN1* gene as ssDNA, leading to bisulfite-induced G-to-A mutations in the *CAN1* sense strand (Figure S7A, left panel). In contrast, transcription of *CAN1* transiently exposes its sense strand as ssDNA, resulting in bisulfite-induced C-to-T mutations in the sense strand (Figure S7A, right panel).

To verify the mutation profiles, we performed PCR amplicon sequencing of the *can1* genes from pools of >1,000 Can^R^ colonies. The experiments were conducted using the Sag1_sg1&sg2 and Sag1_sgTEF1 strains under conditions that induced both d*Sp*Cas9 binding and DSB formation. The results showed that the predominant mutation type at *can1* in the Can^R^ clones from both strains was G-to-A transitions (Figure S7B). This finding confirmed that ssDNA formation during end resection, but not transcription, combined with the mutagenic effects of bisulfite, was the primary source of mutations leading to the generation of Can^R^ mutants. The distribution of G-to-A mutations in the target region (Figure S7C) revealed that mutations were dispersed throughout the *can1* gene, rather than being concentrated at specific sites.

Taken together, these results validate the concept of controlled kataegis, albeit with moderate efficiency. Blocking end resection with d*Sp*Cas9 has potential applications in regionally limited random mutagenesis by DSB induction in the presence of ssDNA-damaging agents.

## Discussion

The primary goal of this study was to determine whether d*Sp*Cas9 can attenuate DSB end resection *in vivo*, with the aim of enabling unique applications of end resection blockage, including controlled kataegis. To achieve this goal, we used two different methods: the ssDNA-specific qPCR assay and ssDNA-specific live cell imaging. Additionally, we performed Southern blot hybridization analysis to confirm stalled end resection at the d*Sp*Cas9-binding sites.

Three sgRNAs (sg1, sg2, and sg3) were used to evaluate the resection-blocking efficiency of d*Sp*Cas9. Both sg1 and sg2 demonstrated ∼50% blocking efficiency, whereas sg3 exhibited a lower blocking efficiency compared to sg1 and sg2 (Figure 1E). This difference is likely due to variations in the binding efficiency of d*Sp*Cas9 to the target DNA, which can be influenced by the accessibility of the target DNA to the d*Sp*Cas9-sgRNA complex, as well as the stability (strength) and residence time of the tertiary complex (i.e., the d*Sp*Cas9-sgRNA complex bound to its target DNA).^32^^,^^33^^,^^47^^,^^48^

The qPCR assay is a gold standard that has been successfully applied to both yeast and human cells.^31^^,^^49^ It offers high sensitivity and specificity for the region of interest. However, it is laborious in practice and cannot distinguish between ssDNA derived from end resection in living cells and degraded ssDNA released from dead cells.

On the other hand, the ssDNA-specific live-cell imaging assay is based on the assembly of the heterotrimeric RPA complex, including Rfa1, on the ssDNA generated by end resection. This assay is simple and rapid, provided that a suitable microscopy system is available. In fact, we routinely use it for the successful selection of effective sgRNAs/crRNAs for genome editing (in preparation). However, it is important to note that the fluorescence signal is not specific to the locus of interest and will inevitably include signals from any DSBs. In addition, the fluorescence intensity of Rfa1-mNeonGreen may not accurately reflect the extent of end resection because Rad52 gradually replaces the ssDNA-bound RPA complex with Rad51 over time. The intensity reflects the balance between the progression of end resection and the displacement of RPA from ssDNA, a dynamic process currently unpredictable and therefore uncorrectable.

Nevertheless, these two assays consistently demonstrate that d*Sp*Cas9 attenuates the progression of end resection *in vivo* (Figures 1 and 2): the results of the two assays obtained from the same cell population correlate well (Figure 3).

It seems counterintuitive that the relative fluorescence intensity in mNG_Sag1_sg1&sg2 was 46% or below 50% (Figure 3C), even though we blocked end resection only on the telomeric side of the DSB with an efficiency of 69% (Figure 3B). Why did this happen? We should note that a Ty2 element located 10 kb upstream of the DSB may limit the expansion of ssDNA, as a previous study showed that Ty elements block end resection.^19^ Considering the asymmetry in end resection, with limited end resection toward the centromeric side but not the telomeric side, we can better explain the relationship between the blocking efficiency and fluorescence intensity.

Because d*Sp*Cas9 must remain bound to its target site until the end resection machinery arrives, its residence time is critical for the successful blockage. Therefore, simultaneous targeting of d*Sp*Cas9 to multiple sites should reduce the likelihood of no d*Sp*Cas9 binding to the region of interest upon the arrival of the end resection machinery, thereby increasing blocking efficiency within the cell population. Indeed, double targeting of d*Sp*Cas9 significantly improved efficiency compared to single targeting (Figure 1E), although complete blockage was not achieved in this study. The magnitude of this improvement appeared to be consistent with the independence of the two blockage events. Besides the multiple targeting strategy, it would also be important to identify and use d*Sp*Cas9 variants or catalytically inactive Cas proteins from other species with longer residence time and/or higher binding strength than d*Sp*Cas9 to improve blockage efficiency.

It is well known that d*Sp*Cas9 can act as a blocker of transcriptional elongation *in vivo* and is thus used as a tool for silencing gene expression. Notably, d*Sp*Cas9 exhibits polarity in blocking transcription: it effectively blocks the transcription machinery approaching from the PAM-proximal side, but not that approaching from the PAM-distal side because the latter disrupts the R-loop.^50^ In contrast, our previous study has shown that d*Sp*Cas9 impairs the progression of replication fork in an orientation-independent manner.^29^ The results of the current study indicate that d*Sp*Cas9 can equally block the end resection machinery approaching from either side (Figures 1C–1E), similar to its effect on the replication fork.

The d*Sp*Cas9-mediated attenuation of end resection led us to consider applying it to regionally restricted random mutagenesis, which we termed controlled kataegis. This concept stems from previous yeast studies that recapitulated kataegis, a mutational storm observed in cancer genomes. In these studies, DSB induction in the presence of ssDNA-damaging agents led to mutagenesis in the DSB-flanking regions, which had been converted to ssDNA by end resection. If d*Sp*Cas9 can limit the progression of end resection, it may also restrict the spatial spread of mutations, providing a unique method for regional random mutagenesis. Considering the extent of end resection, this approach could offer a method for broad region-wide random mutagenesis that is missing from the current toolbox for mutagenesis. We demonstrated the concept of controlled kataegis using the *CAN1* reporter gene. Placing d*Sp*Cas9 between the DSB and the *CAN1* gene resulted in a decrease in Can^R^ mutations induced by DSB formation in the presence of bisulfite (Figure 4).

It is interesting to note that a recent study reported seemingly contradictory results to ours: proximal binding of d*Sp*Cas9 to DSBs stimulates HR, which requires end resection, by inhibiting c-NHEJ in mammalian cells.^51^ The difference between these studies is likely due to the distance between the DSB and the d*Sp*Cas9 binding site. Their study targeted d*Sp*Cas9 within a few hundred bp from the DSB, whereas our study targeted d*Sp*Cas9 over a thousand bp from the DSB. In their study, it is conceivable that d*Sp*Cas9 efficiently inhibits the binding of Ku proteins, the initiator of c-NHEJ, but is readily displaced by the MRN (Mre11-Rad50-Nbs1)–CtIP complex, the initiator of end resection. These events should occur within the region 100–200 bp from the DSB. By contrast, the relevant end resection machinery in our study operates at a distance of over 1 kb from the DSB. Thus, it is not the MRX-Sae2 complex (the yeast equivalent of the MRN-CtIP complex) but rather Exo1 or the STR (Sgs1-Top3-Rmi1)–Dna2 complex that extends end resection. These proteins are likely to be more susceptible to blockage by d*Sp*Cas9. Although it remains to be determined in the yeast whether d*Sp*Cas9 can stimulate HR when targeted to the vicinity of a DSB site, modulating the DSB repair process with d*Sp*Cas9 would be an intriguing area for future research.

In conclusion, we have demonstrated that d*Sp*Cas9 attenuates the progression of end resection *in vivo*. Our findings provide the foundation for a novel method of region-restricted random mutagenesis. We also anticipate that d*Sp*Cas9 will offer researchers a unique tool for mechanistic studies, enabling modulation of the progression of end resection, which is a critical determinant of DSB repair pathways.

### Limitations of the study

The d*Sp*Cas9-mediated blockage of end resection achieved in this study was not complete. We cannot predict the performance of d*Sp*Cas9 targeted to a given genomic site in blocking end resection. It remains to be seen whether, and to what extent, modifications to d*Sp*Cas9 can enhance its ability to block end resection. Therefore, although the concept of controlled kataegis has been proven, it requires further optimization in practice. Additionally, the performance of d*Sp*Cas9-mediated blockage in organisms other than *S. cerevisiae* needs to be investigated in future studies.

## Resource availability

### Lead contact

Further information and requests for resources and reagents should be addressed to and will be fulfilled by the lead contact, Takashi Ito (ito.takashi.352@m.kyushu-u.ac.jp).

### Materials availability

Requests for the generated plasmids and strains in this study should be directed to the lead contact, Takashi Ito (ito.takashi.352@m.kyushu-u.ac.jp).

### Data and code availability

• All raw sequencing data used in this study were deposited in NCBI SRA: SRX27122169, SRX27122170, SRX27122171, SRX27122172, SRX27122173, SRX27122174.
• All original codes used in this study are available from Zenodo at https://doi.org/10.5281/zenodo.14557369.

## Acknowledgments

We are grateful to Hiroaki Takesue and Yuki Sugiyama for their valuable discussions. We thank the technical support from the Research Support Center of the Research Center for Human Disease Modeling at Kyushu University Graduate School of Medical Sciences, which is partially supported by the Mitsuaki Shiraishi Fund for Basic Medical Research. This work was supported by JST CREST Grant Number JPMJCR19S1 and JSPS KAKENHI Grant Number 24K02015.

## Author contributions

Conceptualization, S.T., S.O., and T.I.; funding and resources, T.I.; data production, analyses, investigation, and visualization, S.T. and S.O.; writing, S.T., S.O., and T.I.

## Declaration of interests

The authors declare no competing interests.

## Declaration of generative AI and AI-assisted technologies

During the preparation of this work, the authors used ChatGPT to improve readability of some sentences. After using this tool or service, the authors reviewed and edited the content as needed and take full responsibility for the content of the publication.

## STAR★Methods

### Key resources table

**Table:** 

REAGENT or RESOURCE	SOURCE	IDENTIFIER
**Bacterial and virus strains**		

DH5α high Champion^TM^ cell	SMOBIO	Cat# CC5202

**Chemicals, peptides, and recombinant proteins**		

D-(+)-Raffinose pentahydrate	FUJIFILM Wako Pure Chemical	Cat# 17629-30-0
D-(+)-Galactose	Nacalai Tesque	Cat# 16550-65
Sodium bisulfite	KANTO CHEMICAL	Cat# 7631-90-5
L-Canavanine sulfate	Nacalai Tesque	Cat# 07019-54
Doxycycline hydrochloride	Apollo Scientific	Cat# BID0121

**Critical commercial assays**		

KOD One® PCR Master Mix (Dye-free 2×PCR Master Mix)	TOYOBO	Cat# KMM-101
KOD SYBR® qPCR Mix	TOYOBO	Cat# QKD-201
TB Green Premix Ex Taq II (Tli RNaseH Plus)	TAKARA BIO	Cat# RR820W
Chelex 100 Chelating Resin, biotechnology grade, 100–200 mesh, sodium form	Bio-Rad	Cat# 1432832
Quick-DNA Fungal/Bacterial Miniprep Kit	Zymo Research	Cat# D6005
NEB Golden Gate Assembly Kit (BsaI-HF v2)	New England Biolabs	Cat# E1601L
NEBuilder HiFi DNA Assembly Master Mix	New England Biolabs	Cat# E2621L
Wizard® SV Gel and PCR Clean-Up System	Promega	Cat# A9285
Gene-Packman Coprecipitant kit	Nacalai Tesque	Cat# 12680-30
6×Alkaline Agarose Gel Loading Dye	Thermo Fisher Scientific	Cat# J62157.AC
Agarose for ≥1 kbp fragment, Fine Powder	Nacalai Tesque	Cat# 02468-24
Nytran SPC, 0.45 μm Nylon Transfer Membrane	Cytiva	Cat# 10416230
AlkPhos Direct Labelling Module for 25 labellings	Cytiva	Cat# RPN3680
CDP-Star Detection Reagent for 2,500 cm^2^ membrane	Cytiva	Cat# RPN3682
AlkPhos Direct Hybridization Buffer for 5,000 cm^2^ membrane	Cytiva	Cat# RPN3688
Monarch RNase A	New England Biolabs	Cat# T3018L
SphI-HF	New England Biolabs	Cat# R3182L
MluI-HF	New England Biolabs	Cat# R3198L
HIndIII-HF	New England Biolabs	Cat# R3104S
QIAGEN Genomic DNA Buffer Set	QIAGEN	Cat# 19060
Zymolyase	Zymo Research	Cat# E1005
Protease	QIAGEN	Cat# 19155

**Deposited data**		

Raw sequence data	This paper	NCBI SRA: SRX27122169, SRX27122170, SRX27122171, SRX27122172, SRX27122173, SRX27122174

**Experimental models: Organisms/strains**		

*S*. *cerevisiae*: Strain background: BY4742	Brachmann et al.^52^	
Yeast Deletion Clones *MAT***a** Complete Set	Invitrogen	Cat# 95401.H2
All other synthetic yeast strains used in this paper, listed in Table S1	This paper	N/A

**Oligonucleotides**		

All oligonucleotides used in this paper, listed in Table S2	This paper	N/A

**Software and algorithms**		

ImageJ/Fiji	Schindelin et al.^53^	https://imagej.net/software/fiji/
Bowtie2 v2.2.4	Langmead et al.^54^	https://github.com/BenLangmead/bowtie2
samtools v1.6	Li et al.^55^	https://github.com/samtools/samtools
IGVtools v 2.16.2	Robinson et al.^56^	https://igv.org/
ImageJ script for slice selection	This paper	https://doi.org/10.5281/zenodo.14557369

**Other**		

All plasmids used in this paper, listed in Table S2	This paper	N/A

### Experimental model and subject details

The budding yeast *Saccharomyces cerevisiae* was used as the primary experimental model in the study. The haploid yeast strain BY4742 was used as the parental strain.

### Method details

#### Yeast strains

The yeast strains used in this study are listed in Table S1. Every strain employed in this investigation is a derivative of *S. cerevisiae* YIT9179 (*MAT*α *his3*Δ1 *leu2*Δ0 *lys*2Δ0 *ura3*Δ0 *cup1*Δ::*KanMX*) derived from BY4742 (*MAT*α *his3*Δ1 *leu2*Δ0 *lys2*Δ0 *ura3*Δ0).^52^ This derivation was achieved by deletion of the *CUP1* array and integrating the MscI-cut plasmid containing the *KanMX* cassette flanked by the partial DNA segments of *CIC1* and *RSC30* (Tables S1 and S2). Standard culture media and genetic methods were used in this study.^57^

All the strains used in this study have the common genotype of *ade2*Δ*::HphMX*, *can1*Δ0, insertion of an *ADE2-CAN1* cassette, and insertion of pCSE4-d*Sp*Cas9. The *ADE2* gene was replaced by the *HphMX* cassette using the PCR-based method.^58^ The *CAN1* gene was deleted by genome editing with a genome editing plasmid (*URA3*, *CEN*) (Table S2) as previously described.^59^ The *ADE2-CAN1* cassette was integrated into the *his3*Δ1 locus on chromosome XV using a genome editing plasmid (Table S2) as described previously.^59^ The integrated DNA segment was a PCR product amplified from a plasmid listed in Table S2 using the primers listed in Table S3. The gene encoding d*Sp*Cas9 under the control of the *CSE4* promoter was integrated into a locus between *CIC1* and *RSC30* on chromosome VIII by genome editing or plasmid integration. To construct *rad51*Δ strains, we amplified a fragment containing the *KanMX* cassette flanked by the upstream and downstream sequences of *RAD51* from the *rad51*Δ strain in the Yeast Deletion Clones *MAT***a** Complete Set (Invitrogen) and used it to disruptively replace the *RAD51* gene in our strain background.

The strains containing the gene of enhanced *As*Cas12a (en*As*Cas12a),^60^ *Sa*Cas9, or Rfa1-mNeonGreen fusion protein were constructed by the plasmid integration.

All plasmids and primers used for yeast strain construction are listed in Tables S2 and S3. The sgRNAs and crRNAs for genome editing are listed in Table S4. The strains containing sgRNAs/crRNAs were constructed by transformation of the centromeric plasmids containing genes encoding sgRNAs/crRNAs.

#### Plasmids

All plasmids used in this study are listed in Table S2. All primers for plasmid construction were purchased from Sigma-Aldrich and Eurofins Genomics. Plasmids were constructed with seamless cloning with HiFi DNA Assembly or Golden Assembly purchased from New England Biolabs (NEB).

The integrative plasmids YIplac128-pGAL1-yenAsCas12a-tADH1 (*LEU2*) and YIplac128-pGAL1-y*Sa*Cas9-tADH1 (*LEU2*) contain a gene encoding en*As*Cas12a^60^ or *Sa*Cas9 fused with SV40 nuclear localization signal (NLS)^61^ under the control of the *GAL1* promoter. Both plasmids were used for yeast transformation after AgeI digestion to be integrated into the *GAL1* promoter on the genome.

The integrative plasmid YIpHIS-pCSE4-dCas9-tADH1 (*HIS3*) harbors a gene encoding d*Sp*Cas9 fused with the SV40 NLS^61^ under the control of the *CSE4* promoter. The plasmid was used for yeast transformation after NruI digestion to be integrated into the *CSE4* promoter on the genome.

The integrative plasmid YIpHIS-RFA1C-ymNeonGreen-tADH1 harbors a gene fragment encoding a C-terminal portion of Rfa1 fused with the gene encoding a bright yellow-green fluorescent protein mNeonGreen^36^ at the 3′-end of *RFA1*. The linker sequence between the Rfa1 and mNeonGreen protein is RIPGLINS. The plasmid was used for yeast transformation after MfeI digestion to be integrated into the gene of *RFA1* on the genome.

Centromeric plasmids (*URA3*) for the expression of sgRNAs/crRNAs contain a single sgRNA/crRNA gene, designed to induce a DSB under the control of the *GAL1* promoter, as well as one or two sgRNA genes for d*Sp*Cas9 targeting under the control of the *TetOn-3G* promoter (Tables S2 and S4). Each sgRNA/crRNA gene is flanked by hammerhead and HDV ribozymes to enable autonomous excision from the primary transcript.

For designing sgRNAs for d*Sp*Cas9 and *Sa*Cas9, CRISPRdirect^62^ was used to select target sequences. For designing crRNAs for en*As*Cas12a, CRISPOR^63^ was used to select target sequences. All sgRNAs and crRNAs used in this study are listed in Table S4.

#### Cell culture

Frozen stock cells were woken up on a SC−Ura agar plate at 30°C and inoculated in 2 mL of SC−Ura medium with 3% raffinose for pre-culture at 30°C with 250 rpm rotation. The OD_600_ of overnight culture was measured, and a specific volume was taken to inoculate 20 mL to 50 mL of SC−Ura medium with 3% raffinose and 10 μg/mL Dox to achieve an initial OD_600_ of 0.01. This culture was incubated at 30°C with 250 rpm rotation for approximately 18 h until reaching a final OD_600_ of about 0.5–1.0. Subsequently, Dox was added to a final concentration of 25 μg/mL, and Gal or glucose was added to a final concentration of 2%. The culture was continued at 30°C with 250 rpm rotation. Dox was used to induce the expression and binding of d*Sp*Cas9. Gal was used for DSB induction. Glucose served as a control for no DSB induction in the qPCR assay. After DSB induction, the cells were harvested for DNA extraction to perform ssDNA-specific qPCR assay, subjected to live-cell imaging assay, or treated by bisulfite for genetic assay.

#### SphI digestion for ssDNA-specific qPCR

The genomic DNA was extracted from the harvested cells with Quick-DNA Fungal/Bacterial Miniprep Kit (ZYMO RESEARCH). The concentration of extracted genomic DNA was measured with Qubit dsDNA BR assay on Qubit Flex Fluorometer (Thermo Fisher Scientific), and the DNA solution was diluted to the concentration of 10 ng/μL. Each SphI-digestion solution (25 μL) contained 12.5 μL of 10 ng/μL genomic DNA, 1 μL of SphI-HF (NEB), 2.5 μL of 10×CutSmart buffer. The mock-digestion solution (25 μL) was the same as the SphI-digestion solution, except that SphI-HF was replaced with distilled water. The final concentration of genomic DNA in SphI- or mock-digestion (no SphI digestion) solution was 5 ng/μL. The digestion was performed at 37°C for 3 h–6 h.

#### qPCR

The DNA or SphI/mock-digestion solution was diluted to 1 ng/μL before qPCR. The primers utilized for qPCR are detailed in Table S3. Each qPCR assay was conducted in duplicate, employing QuantStudio3 (Applied Biosystems) following the manufacturer’s instructions.

For amplicons of SphI-1, SphI-2, and SphI-3, each qPCR solution (20 μL) consisted of 2 μL of DNA (2 ng), 10 μL of TB Green Premix Ex Taq II (Tli RNaseH Plus) (Takara), 0.4 μL of ROX Reference Dye II, and 2 pmol each of the forward and reverse primers. The amplification condition involved an initial denaturation at 95°C for 20 s, followed by 40 times iteration of a 3-step thermal cycle comprising 95°C for 10 s, 55°C for 30 s, and 72°C for 10 s. All qPCR runs included 10-fold serial dilutions from an initial concentration of 2 ng/μL or 20-fold dilutions from an initial concentration of 5 ng/μL to generate standard curves.

For amplicons of DSBs including DSB (Sag1), DSB (Sag2), DSB (cr1), and DSB (cr2), each qPCR solution (20 μL) contained 2 μL of DNA (2 ng), 10 μL 2×KOD SYBR qPCR Mix (TOYOBO), 0.4 μL of 10-fold diluted 50×Reference dye, and 2 pmol each of the forward and reverse primers. The amplification condition involved an initial denaturation at 98°C for 20 s, followed by 40 cycles of a 3-step thermal cycle comprising 98°C for 10 s, 56°C for 30 s, and 68°C for 12 s. All qPCR runs included 10-fold serial dilutions from an initial 5 ng/μL concentration to generate standard curves.

The quantity of all amplicons, including SphI-1, SphI-2, SphI-3, and the four DSB amplicons, was normalized to that of *ACT1*.

#### Resection efficiency calculation from the measurements of qPCR

To calculate the end resection efficiency at the specific SphI sites (SphI-1, SphI-2, and SphI-3) after a defined period of DSB induction, we used the following formula, a modified version of the one introduced by Ferrari et al.^31^:

$$r=\frac{2\left(D-1+e\right)}{f\left(D-1+2e\right)}$$

In this formula, r represents the end resection efficiency; D represents the fold-difference at a specific SphI site between the SphI- and mock-digested samples; e represents the efficiency of SphI digestion, determined by subtracting the fold-difference at a specific SphI site between the SphI- and mock-digested samples, which had no DSB induction, from 100% (note that when e is 1, the equation is identical to the original one^31^); f represents the frequency of DSB formation at each target site. The value of f is calculated by subtracting the fold-difference between the test and control samples at the specific cleavage site from 100%. The samples used for measuring DSB formation frequency are those not digested with SphI. All fold differences were calculated based on the Comparative Quantitative Algorithm Calibration Curve Method (CQACCM) of qPCR.^64^ All cycle threshold (Ct) values used in calculating fold difference were averages from duplicate measurements in qPCR reactions.

#### Normalization for resection efficiency

We divided the resection efficiency at each SphI site by that at SphI-1 in the same sample to allow comparisons between different samples and reactions. To eliminate the variance introduced by the different distances from the DSB to the three SphI sites, we divided the resection efficiency at each SphI site in the sample by that at the corresponding SphI site in the DSB reference sample with d*Sp*Cas9 targeting *TEF1*.

#### Southern blot hybridization

Genomic DNA was isolated from cell pellet (approximately 3 × 10^8^ to 10 × 10^8^ cells per sample) resuspended in 1 mL of Buffer Y1 (from QIAGEN Genomic DNA Buffer Set). The suspension was supplemented with 100 units of Zymolyase (Zymo Research) and incubated at 30°C for at least 30 min. The resulting spheroplasts were pelleted by centrifugation at 5,000 × g for 10 min at 4°C. The spheroplast pellet was resuspended in 2 mL of Buffer G2 (from QIAGEN Genomic DNA Buffer Set) containing 0.2 mg/mL RNase (NEB), and 45 μL of QIAGEN Protease stock solution (QIAGEN) was added to the suspension. The mixture was incubated at 50°C for at least 30 min until the solution became clear. Cellular debris was pelleted by centrifugation at 5,000 × g for 10 min at 4°C. The supernatant was mixed with an equal volume of Membrane Binding Solution (from the Promega Wizard SV Gel and PCR Clean-Up System) and transferred to a Zymo-Spin II CR column (from Zymo Research Quick-DNA Fungal/Bacterial Miniprep Kit). Genomic DNA was purified according to the kit instructions and eluted into 100 μL of Elution Buffer provided in the kit.

The purified genomic DNA was digested with MluI (NEB) and HindIII (NEB) in a 100-μL reaction mixture containing 5 μL of MluI, 5 μL of HindIII, and 50 μL of gDNA (concentration: ∼20–60 ng/μL). The reaction was carried out at 37°C for 6 h. The MluI-HindIII digested gDNA was concentrated using Gene-Packman Coprecipitant kit (Nacalai Tesque) to a final concentration of ∼80–200 ng/μL.

The digested DNA sample was then mixed with 6×Alkaline Agarose Gel Loading Dye (Thermo Fisher Scientific) and electrophoresed on a 12-cm long, 1% alkaline agarose gel (Nacalai Tesque).^65^ The electrophoresis was conducted at a constant voltage of 20 V for ∼16 h to separate DNA fragments.^65^ The gel was then soaked in neutralization solution (1 M Tris-HCl, pH 7.6, 1.5 M NaCl) for 45 min at room temperature. DNA was transferred onto a nylon membrane (Cytiva) via capillary blotting using the G Capillary Blotter C-set (TAITEC) according to the manufacturer’s protocol. The transfer process was performed overnight. After transfer, the DNA was crosslinked to the membrane using a UVP Crosslinker set to 120 mJ/cm^2^ for 23 s.

Hybridization was performed using a 180-mer probe (Table S3) labeled with the AlkPhos Direct Labeling Module (Cytiva). The hybridization was conducted in a glass bottle in a hybridization oven at 55°C overnight. Post-hybridization washes were performed using primary and secondary wash buffers prepared according to the Amersham Gene Images AlkPhos Direct Labeling and Detection System Product Booklet. The washing procedure was also according to the same manual. After washing, 1 mL of CDP-Star Detection Reagent (Cytiva) was applied to the membrane. Chemiluminescent signals were captured using the ImageQuant 800 system (Cytiva), positioning the membrane in the upper slot and exposing it for 32 min with 5 × 5 binning. The Southern blot hybridization images were saved as 16-bit TIFF files.

For visual presentation, the raw TIFF images were processed using Fiji/ImageJ with the following steps: image size was reduced to 50%; outliers were removed with a radius of 7 pixels and a threshold of 50; background subtraction was performed using a rolling ball radius of 50 pixels; and smoothing was applied.

#### Fluorescence microscopy and image processing

Image acquisitions of yeast cells were performed on a microscope (ECLIPSE Ti-E, Nikon) equipped with a 20× objective lens (CFI Plan Apo λ 20×, MRD00205, Nikon), a sCMOS camera (ORCA-Fusion BT, C15440-20UP, Hamamatsu photonics), and a solid-state illumination light source (SOLA SE II, Lumencor). The image acquisition process was controlled by NIS-Elements version 5.3 (Nikon). The binning mode of the camera was set at 1 × 1 (0.33 μm/pixel), and Z-stacks were acquired at 7 × 0.9 μm intervals. For imaging of Rfa1-mNeonGreen, a YFP filter set (LED-YFP-A, Semrock) was used, with the excitation light power set to 25% and an exposure time of 250 msec/frame. For differential interference contrast (DIC) imaging, the exposure time was 20 msec/frame. DIC images were captured at the central position of the Z-stacks.

Image processing and analysis were performed using Fiji/ImageJ.^53^ To generate images of the YFP channel from Z-stacks, background subtraction was applied using a sliding paraboloid with a radius of 5 pixels and smoothing disabled. The Z slice with the highest sum intensity out of seven was selected as the most focused slice and used for the following quantitative analysis. The ImageJ script for the slice selection is available at https://doi.org/10.5281/zenodo.14557369. The mean of the raw integrated density values of all the particles for each sample was used for the fluorescence intensity quantification. After global adjusting of brightness and contrast and cropping of the images, sequences of representative images were generated.

#### Relative fluorescence intensity calculation

The relative fluorescence intensity calculation is performed according to the formula below:

$$relative\;I=\frac{I-\bar{I_{NC}}}{\bar{I_{DSB}}-\bar{I_{NC}}}$$

In this formula, I represents the fluorescence intensity; $\bar{I_{NC}}$ represents the average fluorescence intensity of the negative control cells, which have neither d*Sp*Cas9 binding nor DSB formation; $\bar{I_{DSB}}$ represents the average fluorescence intensity of the DSB reference cells, which have DSB formation but no d*Sp*Cas9 bound near the intended DSB position.

#### Bisulfite treatment

Dox (final concentration: 25 μg/mL) and Gal (final concentration: 2%) were added into a 20-mL culture of log-phase cells (OD_600_: ∼0.5 to ∼1.3) to induce d*Sp*Cas9-binding and DSB generation. Following a 4-h induction time, the cells were washed twice with 100 mM sodium citrate (pH 5.2) supplemented with 2% Gal and 25 μg/mL Dox. The washed cells were then resuspended in 2 mL of the same buffer. Sodium bisulfite (KANTO CHEMICAL) was added to the half of the cell suspension to a final concentration of 1% for bisulfite treatment, while an equivalent volume of H_2_O was added to the remaining half to serve as a control. Following incubation at 30°C for 2.5 h, the bisulfite-treated and control cells were washed twice with H_2_O and resuspended in 500 μL of H_2_O. Subsequently, 250 μL–450 μL of the suspension was plated onto SC_low-Ade_Can plate (comprising 2% glucose, 10 mg/L adenine, and 60 mg/L canavanine), and 100 μL of 10^5^-fold dilution was spread onto an SC plate (containing 2% dextrose).

#### Mutation frequency calculation

Colony counting on the SC and SC_low-Ade_Can plates was performed to quantify the density of live cells and Can^R^ mutants: the Can^R^ mutation frequency was determined by dividing the latter by the former. Counting of red colonies on the SC_low-Ade_Can plate was employed for a similar calculation of the frequency of dual mutations involving Can^R^ and Ade^−^. To mitigate the impact of varying DSB formation frequencies among different cells on the mutation frequency, the mutation frequency was normalized by dividing it by the DSB formation frequency. For the statistical analysis of mutation frequencies across multiple experiments, we calculated the relative normalized mutation frequencies by dividing the normalized mutation frequencies of the strains by that of the DSB reference strain.

#### Targeted deep sequencing

Colonies grown on SC plates supplemented with canavanine were harvested and resuspended in 1 mL of 15% glycerol. An aliquot of the suspension (5 μL) was used to inoculate 20 mL of SC liquid medium containing canavanine, followed by overnight culture at 30°C with shaking at 250 rpm. Genomic DNA was subsequently extracted from the culture and used as a template for PCR amplification. Two regions were amplified: the *can1* target region (2,192 bp, located on Chromosome XV) and the *ALP1* control region (1,947 bp, located on Chromosome XIV). The *ALP1* region served as a control to monitor the background mutation frequency. The primers used in this experiment are listed in Table S3.

For library preparation, 100 ng of PCR product per sample was processed using the Illumina DNA Prep (M) Tagmentation Kit (Illumina). Deep sequencing was performed by Genome-Lead Inc. on the NovaSeq 6000 platform using the SP Reagent Kit v1.5 (300 cycles) and a 151-cycle × 2 paired-end sequencing strategy. Base calling and generation of FASTQ files for each barcode were conducted using BCL Convert v3.9, with adapter trimming enabled.

The sequencing data generated in this study have been deposited in the NCBI Sequence Read Archive (SRA) under BioProject ID PRJNA1199164.

#### Targeted deep sequencing data analysis

The raw sequencing reads obtained from NovaSeq 6000 were processed using a standard bioinformatics pipeline as follows. High-quality reads (Q > 20) were mapped to the reference sequences of the target regions using bowtie2 (version 2.2.4) with default parameters.^54^ The resulting SAM files were converted to BAM format and sorted using SAMtools (version 1.6).^55^ The BAM files were converted to WIG format. The average coverage for each sample ranged from 34,000 to 65,000. IGVtools (version 2.16.2) was used to calculate the frequency of each base at all positions of interest within the target regions.^56^ The relative mutation frequency at each nucleotide position was calculated using the mutation frequencies obtained from untreated cells as a reference dataset. For this calculation, nucleotide positions with a mutation frequency of zero in the untreated cell dataset were excluded to avoid division by zero and ensure accurate comparisons.

#### Assessment of cell viability

The overall cell density was determined using a hemocytometer. The density of viable cells was calculated by counting the number of colonies on an SC plate, using an appropriate dilution of the original cell suspension. Cell viability was measured by calculating the ratio of viable cell density to total cell density.

### Quantification and statistical analysis

Dunnett’s test, Steel-Dwass’s test, Student’s *t*-test, and Welch’s *t*-test were used to calculate *p* values, as indicated in the figure legends. In general, results were considered statistically significant when *p* < 0.05.

## Supplemental information

Document S1. Figures S1–S7

Table S1. Yeast strains used in this study, related to Method Details and Figures 1–4 and S1–S7

Table S2. Plasmids used in this study, related to Method Details and Figures 1–4 and S1–S7

Table S3. Primers and probe used in this study, related to Method Details and Figures 1–4 and S1–S7

Table S4. sgRNAs and crRNAs used in this study, related to Method Details and Figures 1–4 and S1–S7
