# Supplementary material for: Catalytically inactive Cas9 attenuates DNA end resection: A potential application for region-restricted random mutagenesis
Source: iScience. 2025 May 20;28(6):112702. doi: 10.1016/j.isci.2025.112702 (PMC12164011; doi:10.1016/j.isci.2025.112702)
Supplement: Document S1. Figures S1–S7 [file mmc1.pdf]

iScience, Volume *28*

Supplemental information

Catalytically inactive Cas9 attenuates DNA end resection: A potential application for region-restricted random mutagenesis

Suchin Towa, Satoshi Okada, and Takashi Ito

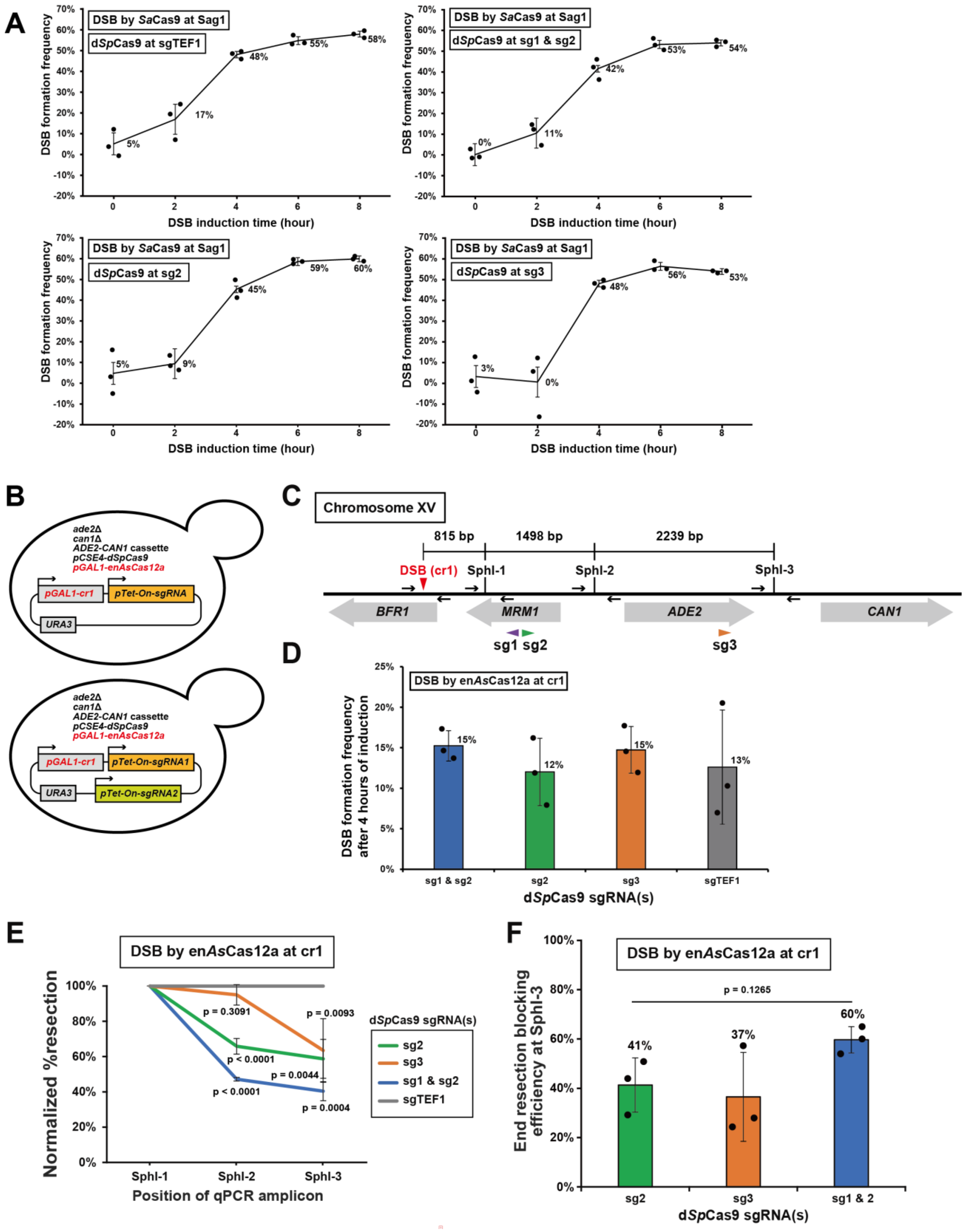

(legend on next page)

Figure S1. ssDNA-specific qPCR to evaluate d*Sp*Cas9-mediated end resection blockage, related to Figure 1

(A) DSB formation frequencies by *Sa*Cas9(Sag1), quantified by qPCR at 0, 2, 4, 6, and 8 hours during continuous DSB induction in four strains, each with a distinct d*Sp*Cas9-bound condition. Data are represented as mean ± standard deviation (SD) (n = 3 biological replicates). Mean value is indicated at each time point, and individual dots represent biological replicates.

(B) Schematic representation of the strains with en*As*Cas12a(cr1) for DSB generation. Expression of en*As*Cas12a and the crRNA cr1 is driven by the *GAL1* promoter, while expression of d*Sp*Cas9 sgRNA(s) is controlled by the *Tet-On* promoter(s).

(C) Schematic representation of the genomic locus used in this study. The DSB site generated by en*As*Cas12a(cr1) is indicated by a red triangle. The target sites of three d*Sp*Cas9 sgRNAs (sg1, sg2, and sg3) are represented by purple, green, and orange arrowheads, respectively. The locations of three qPCR amplicons (SphI-1, -2, and -3), each containing a single SphI site, are shown as black vertical bars accompanied by pairs of horizontal arrows representing the qPCR primers.

(D) DSB formation frequencies by en*As*Cas12a(cr1) in four strains with distinct d*Sp*Cas9-bound conditions, quantified by qPCR 4 hours after DSB induction. Data are presented as mean ± SD (n = 3 biological replicates). Mean values are indicated at the upper right of the bars, and individual dots represent biological replicates for each strain.

(E) Normalized percentage of resection at the three SphI sites in four strains harboring the DSB generated by en*As*Cas12a(cr1), measured at 4 hours after DSB induction. Data are represented as mean ± SD (n = 3 biological replicates). Statistical significance was assessed using Dunnett's test for SphI-2 and SphI-3 sites of the strains harboring sgRNA(s) (sg1 & sg2, sg2, or sg3), compared to the DSB reference strain carrying a control sgRNA (sgTEF1).

(F) End resection blocking efficiency at the SphI-3 site in the three strains with different d*Sp*Cas9 sgRNA(s), measured 4 hours after DSB induction. Data are represented as mean ± SD (n = 3 biological replicates). Mean values are indicated above the bars, and individual dots represent biological replicates for each strain. Statistical significance was assessed using Student's *t*-test for the strain harboring a single sgRNA (sg2), compared to the strain harboring a pair of sgRNAs (sg1 & sg2).

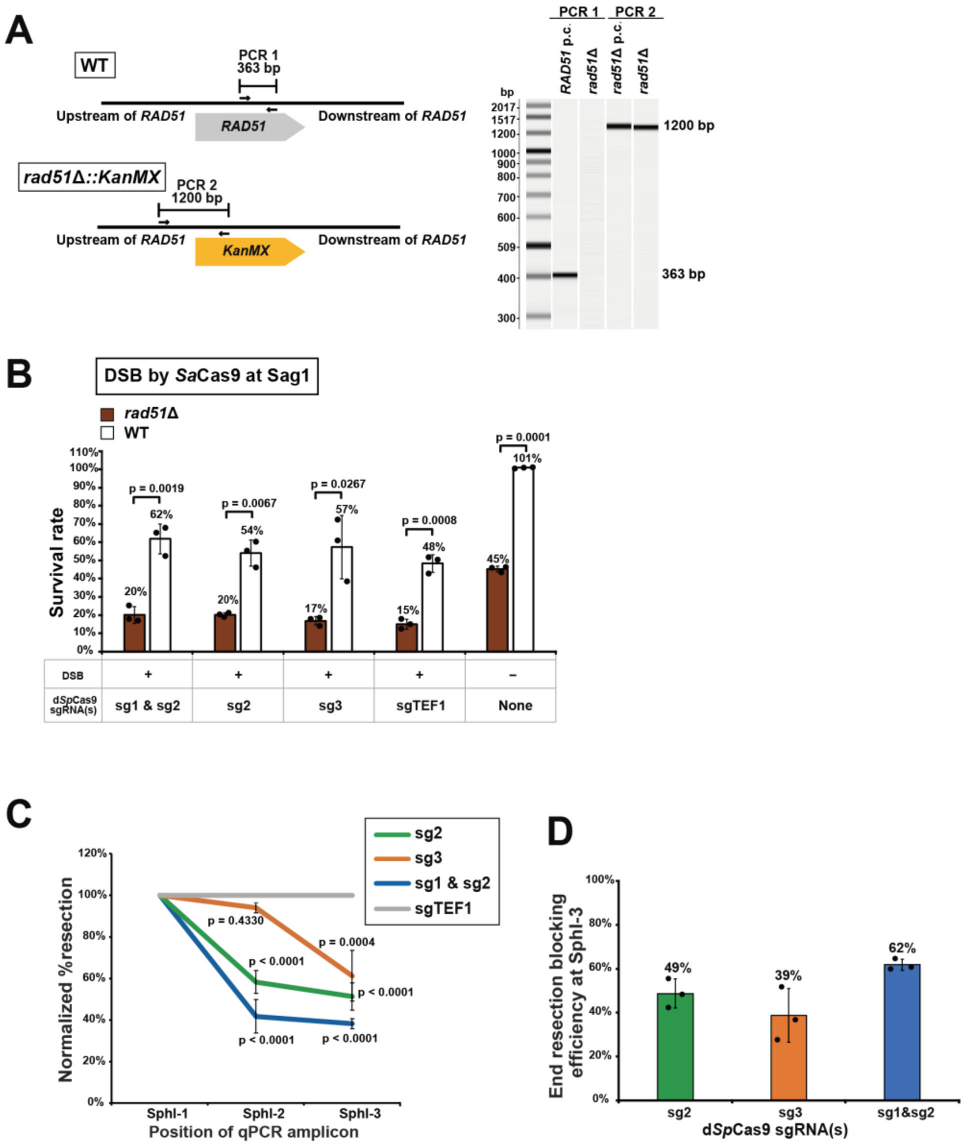

Figure S2. d*Sp*Cas9-mediated end resection blockage in *rad51*Δ cells, related to Figure 1

(A) Genotyping of the *rad51*Δ strain constructed for this experiment. Two PCR assays (PCR1 and PCR2) were performed using the indicated primers (black horizontal arrows), and the amplified products were analyzed with the MultiNA DNA electrophoresis system. The wild-type (WT) strain was used as a positive control for PCR1 (*RAD51* p.c.), confirming the absence of the *RAD51* ORF in the *rad51*Δ strain. The *rad51*Δ*::KanMX* strain from the Yeast Deletion Clones *MAT***a** Complete Set (Invitrogen) served as a positive control for PCR2 (*rad51*Δ p.c.), verifying the precise replacement of the *RAD51* ORF with the *KanMX* cassette.

(B) Survival rate of *rad51*Δ cells, measured 4 hours after DSB induction. Conditions of DSB generation and d*Sp*Cas9 binding are shown below the graph. Data are represented as mean ± SD (n = 3 biological replicates). Mean values are indicated above the bars, and dots indicate individual biological replicates for each strain. Statistical significance between *rad51*Δ and the WT control was evaluated using Welch's t-test.

(C) Normalized percentage of resection at the three qPCR amplicon sites in four strains expressing distinct sgRNA(s), measured 4 hours after DSB induction. The DSB was generated by *Sa*Cas9(Sag1). Data are represented as mean ± SD (n = 3 biological replicates). Statistical significance was assessed using Dunnett's test for the SphI-2 and SphI-3 sites of the strains harboring distinct sgRNA(s) (sg1 & sg2, sg2, or sg3), compared to the DSB reference strain harboring sgTEF1.

(D) End resection blocking efficiency at the SphI-3 site in three strains with distinct d*Sp*Cas9 sgRNA(s), measured 4 hours after DSB induction. The DSB was generated by *Sa*Cas9(Sag1). Data are represented as mean ± SD (n = 3 biological replicates). Mean values are indicated above the bars, and individual dots represent biological replicates for each strain.

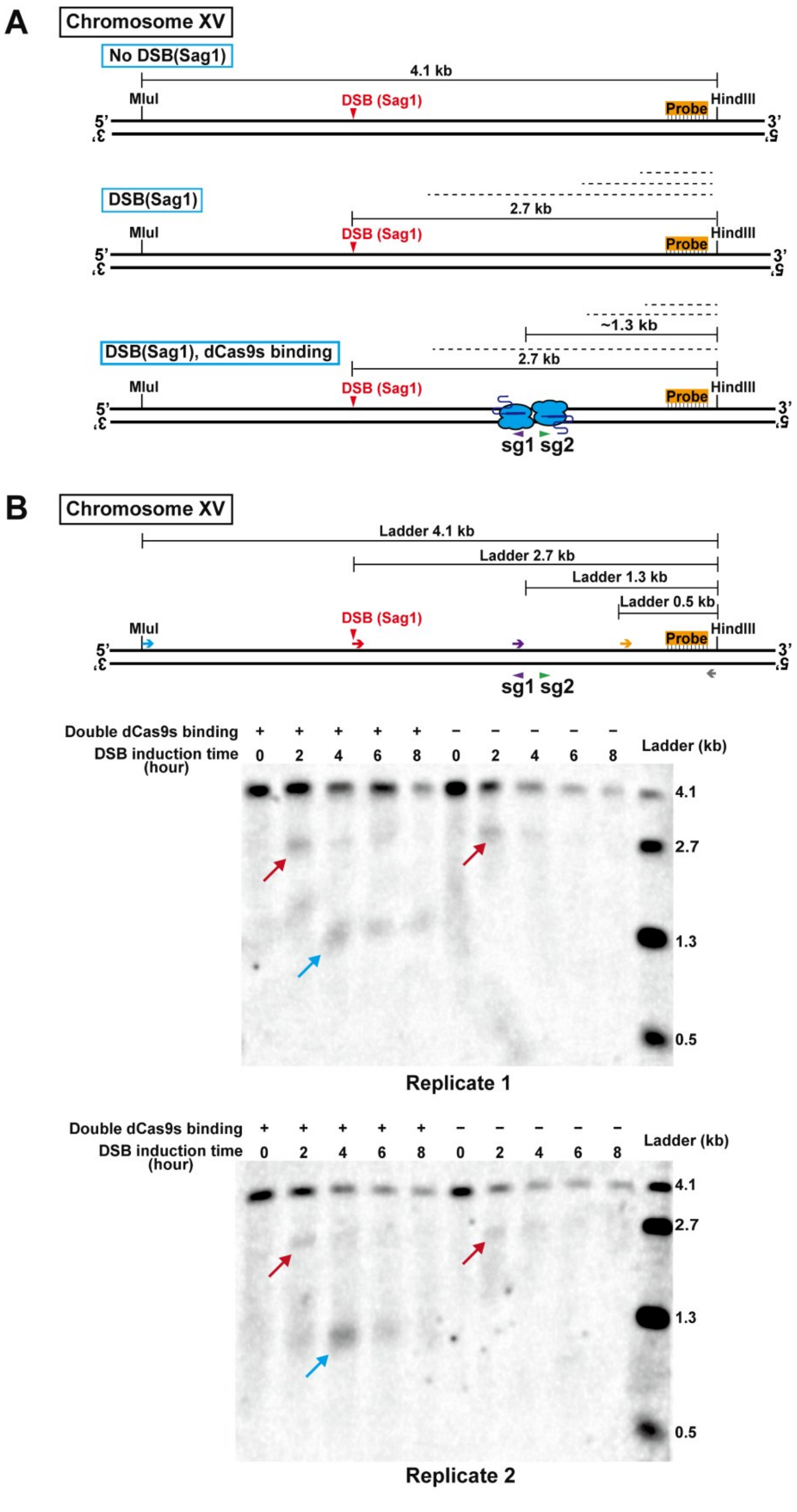

(legend on next page)

Figure S3. Southern blot analysis to confirm d*Sp*Cas9-mediated end resection blockage, related to Figure 1

(A) Schematic representation of experimental design. Solid and dashed lines indicate DNA fragments detected by the 180-mer strand-specific probe. The 4.1-kb fragment represents the intact MluI-HindIII fragment containing the target site of *Sa*Cas9(Sag1). The 2.7-kb fragment represents the *Sa*Cas9(Sag1)-cleaved fragment that has not undergone extensive end resection. The 1.3-kb fragment is expected to appear when end resection is stalled at the binding sites of d*Sp*Cas9. The dashed line represents fragments that are substantially resected but still capable of hybridizing to the probe.

(B) Southern blot hybridization patterns. Upper panel: PCR products used as size standards. The gray leftward arrow represents the reverse primer common to the four PCR products, ranging from 0.5 kb to 4.1 kb. The blue, red, purple, and orange rightward arrows represent the forward primers for the 4.1-kb, 2.7-kb, 1.3-kb, and 0.5-kb PCR products, respectively. Bottom panel: Southern blot hybridization patterns. The red and blue arrows indicate the 2.7-kb and 1.3-kb bands described in (A), respectively.

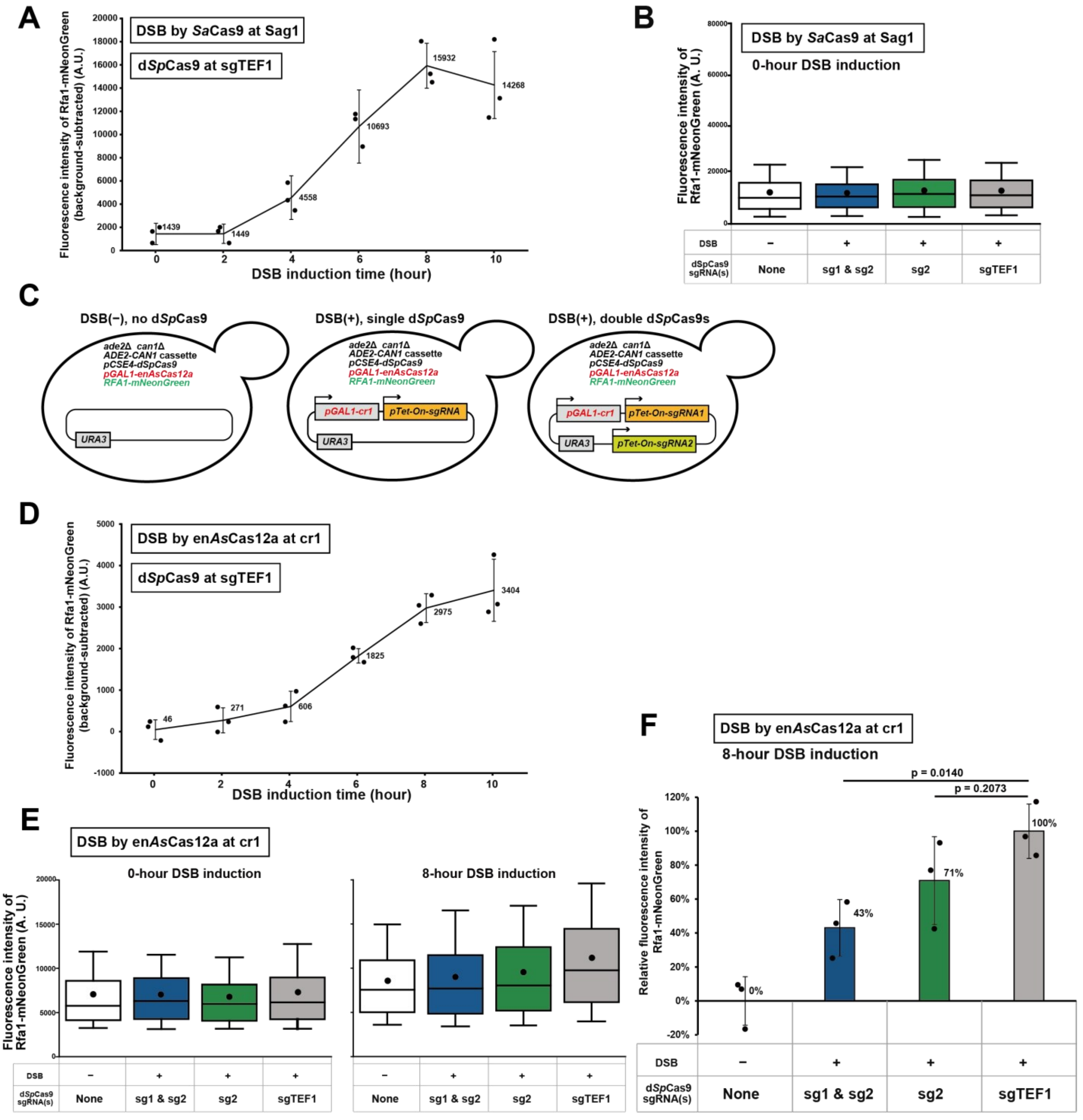

(legend on next page)

Figure S4. Live-cell imaging to assess d*Sp*Cas9-mediated end resection blockage, related to Figure 2

(A) Background-subtracted Rfa1-mNeonGreen fluorescence intensity. Cells harboring sgTEF1 were examined at 0, 2, 4, 6, 8, and 10 hours during continuous DSB induction. The DSB was generated by *Sa*Cas9(Sag1). Data are represented as mean ± SD (n = 3 biological replicates). Mean value is indicated at each time point, and individual dots represent biological replicates.

(B) Box plots showing the distribution of Rfa1-mNeonGreen fluorescence intensity in cells under the indicated conditions. Images were acquired before DSB induction (0-hour DSB induction). Boxes represent the 25th and 75th percentiles, whiskers represent the 10th and 90th percentiles, the horizontal line in the box indicates the median, and the black dot represents the mean.

(C) Schematic representation of the strains with en*As*Cas12a(cr1) for DSB generation. sgRNA indicates a d*Sp*Cas9 sgRNA (sg1, sg2, or sgTEF1).

(D) Background-subtracted Rfa1-mNeonGreen fluorescence intensity in cells harboring sgTEF1 at 0, 2, 4, 6, 8, and 10 hours during continuous DSB induction by en*As*Cas12a(cr1). Data are represented as mean ± SD (n = 3 biological replicates). Mean value is indicated at each time point, and individual dots represent biological replicates for each strain.

(E) Box plots showing the distribution of Rfa1-mNeonGreen fluorescence intensity in cells under the indicated conditions. Images were acquired before and 8 hours after DSB induction. The DSB was generated by en*As*Cas12a(cr1). Boxes represent the 25th and 75th percentiles, whiskers represent the 10th and 90th percentiles, the horizontal line in the box indicates the median, and the black dot represents the mean.

(F) Relative fluorescence intensity in cells with distinct d*Sp*Cas9-bound conditions, measured 8 hours after DSB induction. Data are represented as mean ± SD (n = 3 biological replicates). Mean values are indicated at the upper right of the bars, and individual dots represent biological replicates for each strain. Statistical significance was assessed using Dunnett’s test, comparing strains harboring either a pair of sgRNAs (sg1 & sg2) or a single sgRNA (sg2) with the DSB reference strain harboring sgTEF1.

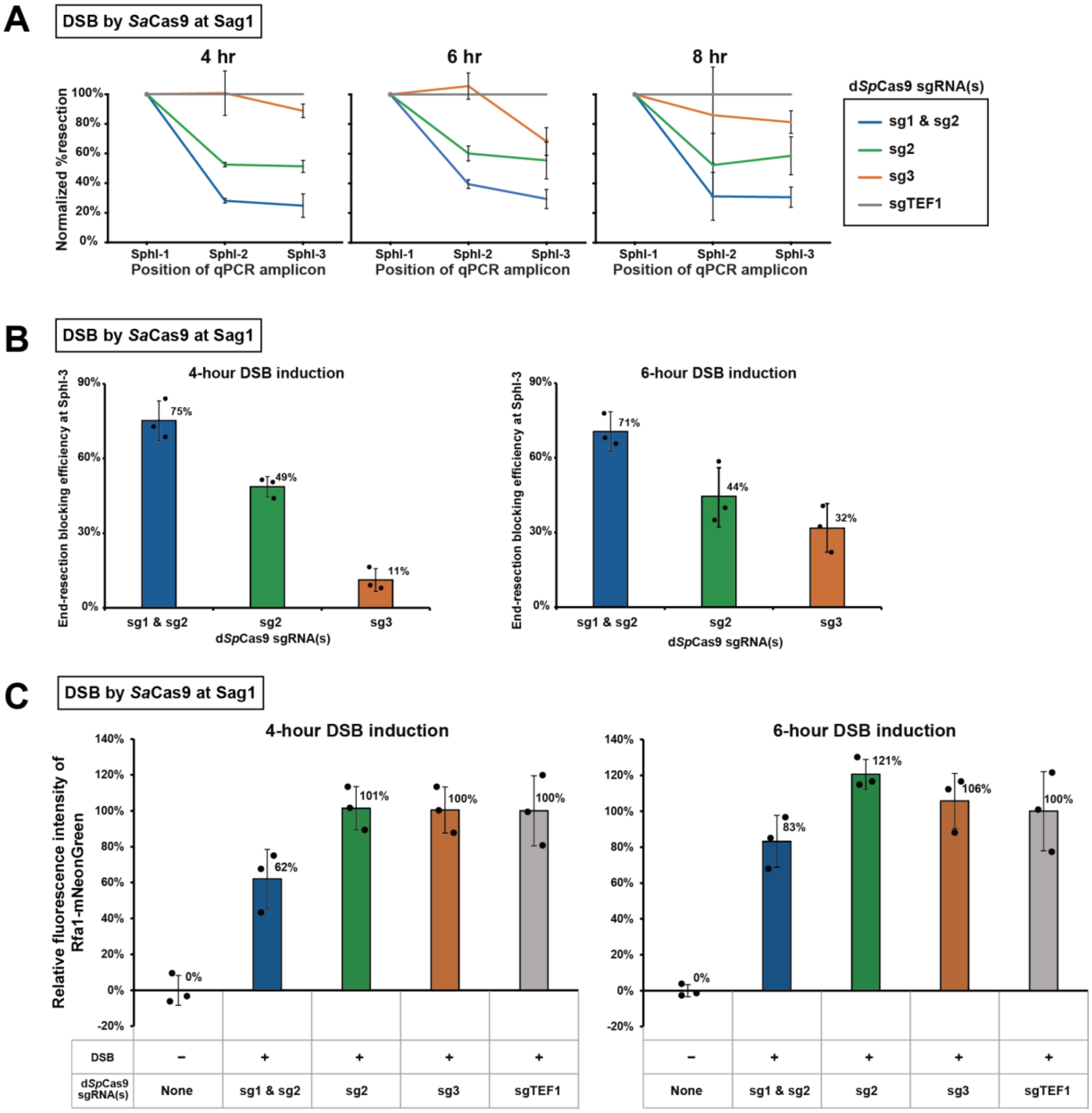

Figure S5. Correlation between end resection blocking efficiency and Rfa1-mNeonGreen fluorescence intensity, related to Figure 3

(A) Normalized percentage of resection at the three SphI sites in the four strains with different d*Sp*Cas9 sgRNA(s), measured at 4, 6, and 8 hours during continuous DSB induction. Data are represented as mean ± SD (n = 3 biological replicates).

(B) End resection blocking efficiency at the SphI-3 site in cells with distinct d*Sp*Cas9 sgRNA(s), evaluated by the ssDNA-specific qPCR assay at 4 and 6 hours during continuous DSB induction by *Sa*Cas9(Sag1). Data are represented as mean ± SD (n = 3 biological replicates). Mean value is indicated at the upper right of the bars, and individual dots represent biological replicates for each strain.

(C) Relative fluorescence intensity of cell populations with the indicated conditions at 4 and 6 hours during continuous DSB induction by *Sa*Cas9(Sag1). Data are represented as mean ± SD (n = 3 biological replicates). Mean values are indicated at the upper right of the bars, and individual dots represent biological replicates for each strain.

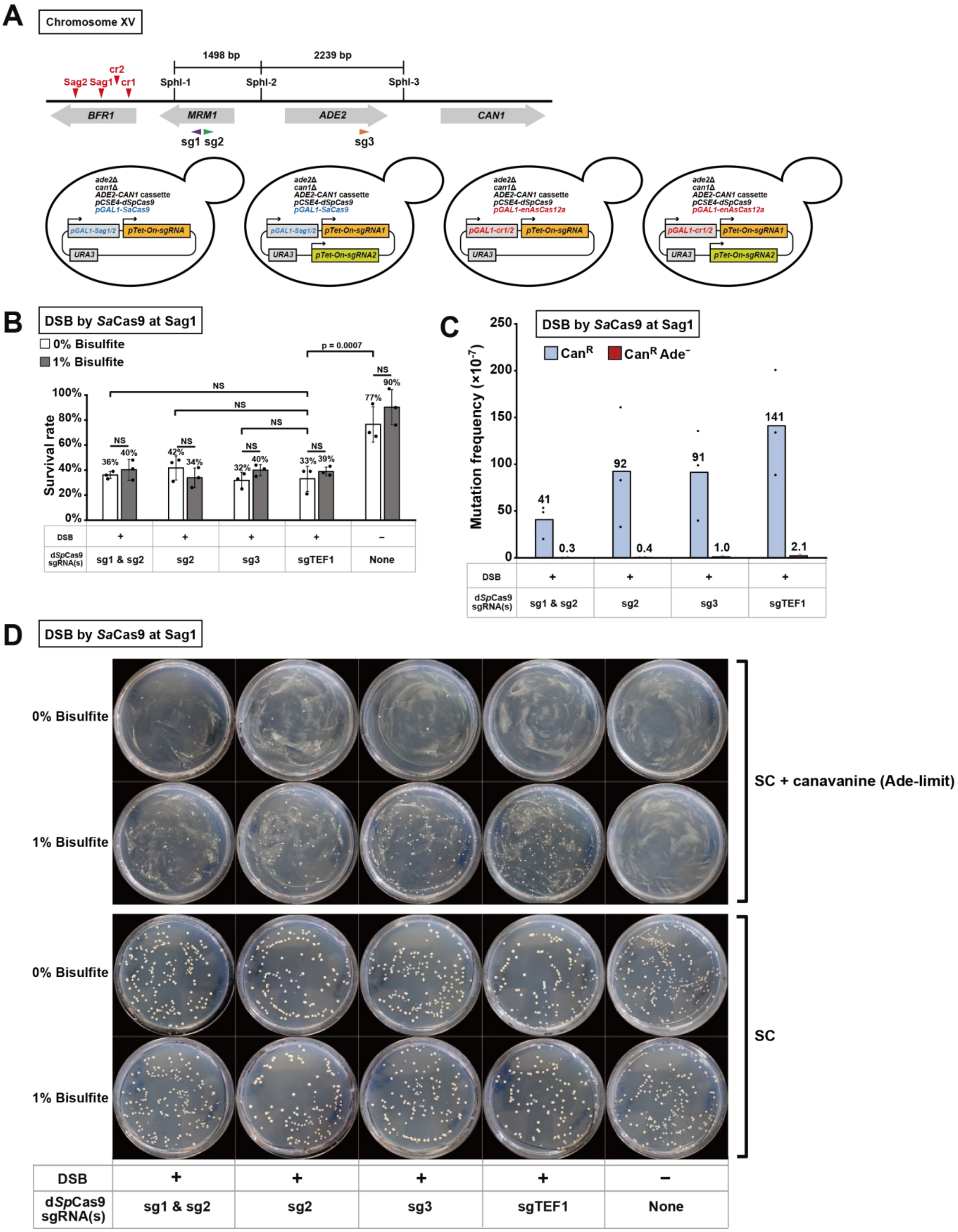

(legend on next page)

Figure S6. Application of d*Sp*Cas9-mediated end resection blockage to region-restricted bisulfite-induced mutagenesis, related to Figure 4

(A) Schematic representation of the genomic locus (top) and the strains (bottom) used in this experiment. Top: Red arrows indicate the positions of DSBs generated by *Sa*Cas9(Sag1 or Sag2) or en*As*Cas12a(cr1 or cr2). Other features are as described in Figure 1C. Bottom: “sgRNA” refers to sg2, sg3, or sgTEF1. “Sag1/2” denotes Sag1 or Sag2, and “cr1/2” indicates cr1 or cr2.

(B) Survival rate of the cells after bisulfite treatment. Conditions for DSB generation and d*Sp*Cas9-targeting are shown below the graph. Data are represented as mean ± SD (n = 3 biological replicates). Mean values are indicated above the bars, and individual dots represent biological replicates for each strain. Statistical significance between the samples treated with bisulfite and the control was assessed using Student’s *t*-test. Statistical significance between samples with and without DSBs and between samples with and without d*Sp*Cas9-mediated end resection blockage was assessed using Dunnett’s test. Each strain was compared to the DSB reference strain harboring sgTEF1.

(C) Mutation frequency after DSB induction in the presence of 1% bisulfite. The DSB was generated by *Sa*Cas9(Sag1). Conditions for DSB generation and d*Sp*Cas9-targeting are described below the graph. The frequencies of Can^{R}, including all canavanine resistance mutations (“Can^{R} only” and “Can^{R} Ade^{-}”), are depicted in light blue. The frequencies of Can^{R} Ade^{-} are shown in red. Mean values are indicated above the bars (n = 3 biological replicates). Individual dots represent biological replicates for each strain.

(D) Representative images of colony formation on SC + canavanine (adenine-limited) and SC agar plates for cells with or without bisulfite treatment and DSB induction.

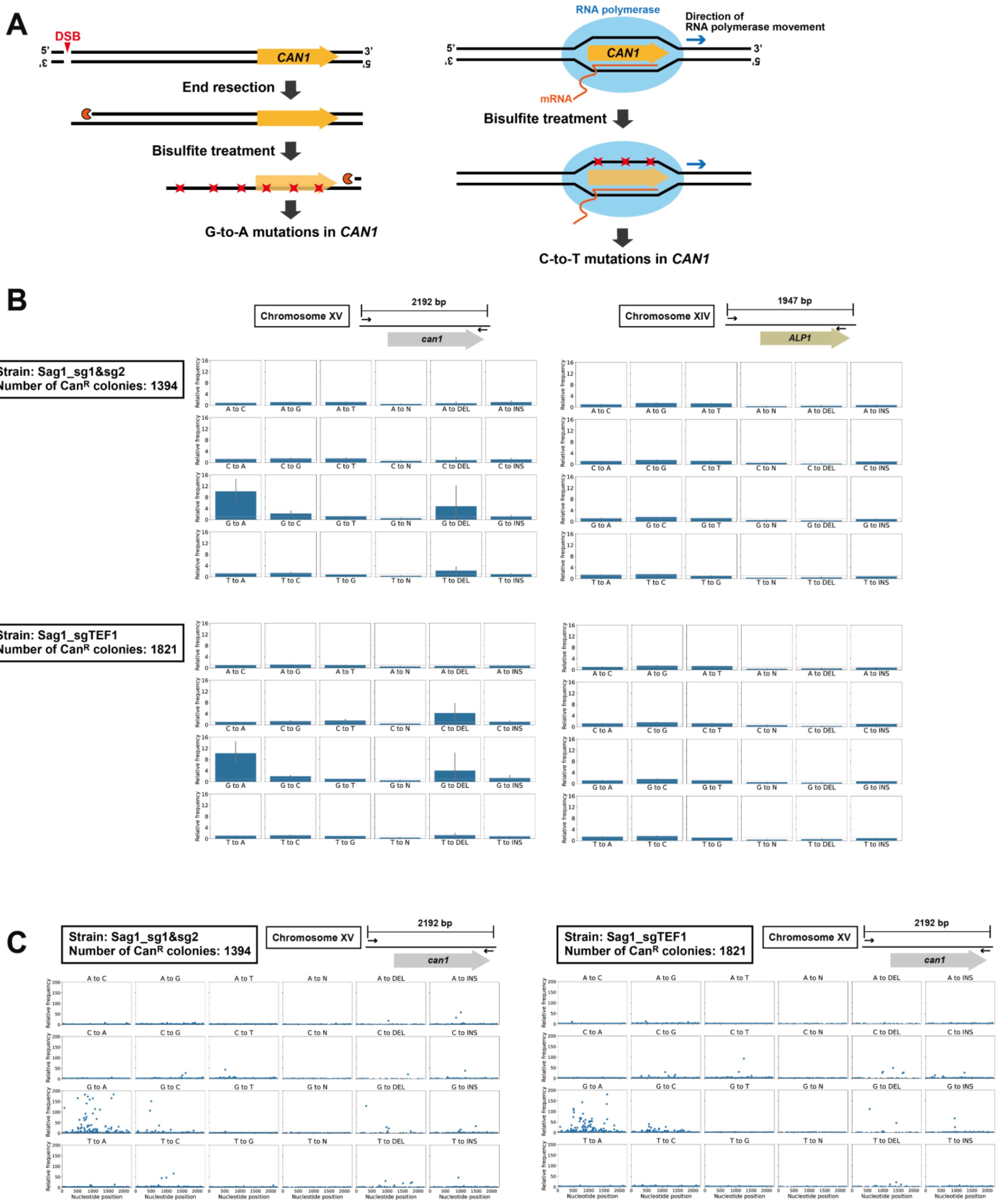

(legend on next page)

Figure S7. Targeted deep sequencing of *can1* gene, related to Figure 4

(A) Schematic representation of bisulfite-induced mutagenesis of the *CAN1* reporter gene. Note that bisulfite-induced deamination upon end resection from the DSB site affects the non-sense (bottom) strand, resulting in G-to-A substitutions on the sense (top) strand (left panel). In contrast, deamination during transcription-coupled R-loop formation occurs on the sense (top) strand, leading to C-to-T substitutions on the sense (top) strand (right panel).

(B) Mutation spectrum in the sense (top) strand of the *can1* and control *ALP1* genes. Mutations are classified into 24 types. DEL and INS indicate deletion and insertion, respectively. Each small panel shows a bar graph representing the average relative frequency of the mutation type indicated at the bottom. Each mutation frequency was normalized to the value observed in untreated cells. Nucleotide positions with zero mutation frequency in untreated cells were excluded from the calculations. The averages represent the mean relative frequencies calculated across all nucleotide positions with available data for each mutation type. Error bars represent 95% confidence intervals. The four 24-panel sets show the results for *can1* in Sag1_sg1&sg2 (top left), *ALP1* in Sag1_sg1&sg2 (top right), *can1* in Sag1_sgTEF1 (bottom left), and *ALP1* in Sag1_sgTEF1 (bottom right). Note that G-to-A substitution is the most prevalent mutation type in *can1*.

(C) Mutation positions in the sense (top) strand of *can1*. Each small panel shows the position (X-axis) and relative frequency (Y-axis) of one of the 24 mutation types indicated at the top of each panel. Frequencies were normalized to those observed in untreated cells, and nucleotide positions with zero mutation frequency in untreated cells were excluded from the calculations. The left 24-panel set shows results from the Sag1_sg1&sg2 strain, while the right 24-panel set shows results from the Sag1_sgTEF1 strain. Note that G-to-A substitutions are distributed throughout the *can1* gene.

Supplemental references

[S1] Brachmann, C.B., Davies, A., Cost, G.J., Caputo, E., Li, J., Hieter, P., and Boeke, J.D. (1998). Designer deletion strains derived from Saccharomyces cerevisiae S288C: a useful set of strains and plasmids for PCR-mediated gene disruption and other applications. Yeast *14*, 115–132. https://doi.org/10.1002/(SICI)1097-0061(19980130)14:2<115::AID-YEA204>3.0.CO;2-2

[S2] Gilbert, L.A., Larson, M.H., Morsut, L., Liu, Z., Brar, G.A., Torres, S.E., Stern-Ginossar, N., Brandman, O., Whitehead, E.V., Doudna, J.A., Lim, W.A., Weissman, J.S., and Qi, L.S. (2013) CRISPR-mediated modular RNA-guided regulation of transcription in eukaryotes. Cell *154*, 442–451. https://doi.org/10.1016/j.cell.2013.06.044
